# Engineered Peptide Self‐Assembled Nanofibers for Enhanced STING Activation: A Promising Nanodelivery Platform for Immunotherapy of Pediatric Solid Tumors

**DOI:** 10.1002/advs.77967

**Published:** 2026-09-27

**Authors:** Yubin He, Xiao Lu, Chang qing Yang, Ran Zhang, Bo Wan, Xin Ni, Dong Mei

**Affiliations:** ^1^ Department of Pharmacy National Center for Children's Health Beijing Children's Hospital Capital Medical University Beijing China; ^2^ School of Basic Medicine and Clinical Pharmacy China Pharmaceutical University Nanjing China; ^3^ National Center for Pediatric Cancer Surveillance National Center for Children's Health Beijing Children's Hospital Capital Medical University Beijing China; ^4^ Clinical Research Ward Capital Center for Children's Health Capital Institute of Pediatrics Capital Medical University Beijing China

**Keywords:** immunotherapy, neuroblastoma, peptide self‐assembled delivery system, pH‐responsive release, STING agonists

## Abstract

STING agonists (e.g., cGAMP), which function as immunotherapeutic agents activating the stimulator of interferon genes (STING) pathway, hold promise for treating pediatric solid tumors including neuroblastoma. However, their clinical translation is hindered by limitations including burst release, short half‐life, poor targeting, and low intracellular delivery efficiency. In this study, we engineered a peptide self‐assembled nanofiber (NFSA) based on the specific interaction mechanism between STING protein and cGAMP. By hydrophobically modifying the cGAMP‐binding amino acid sequences on STING, two amphiphilic peptides, MP1 and MP2, were obtained, which self‐assembled into nanofibers to efficiently encapsulate cGAMP. The NFSA showed excellent biocompatibility and low toxicity both in vitro and in vivo. It enabled pH‐responsive cGAMP release, promoted cellular uptake, enhanced the endoplasmic reticulum (ER) localization, and activated the STING pathway. NFSA also promoted dendritic cell maturation and shifted tumor‐associated macrophages to the anti‐tumor M1 type. In vivo, NFSA reduced neuroblastoma growth, increased CD8^+^ T cell infiltration, induced tumor cell apoptosis, and demonstrated good biosafety. Our study developed a novel, easily producible, and low‐toxicity STING agonist nanodelivery platform, which effectively overcomes key pharmacokinetic and pharmacodynamic barriers limiting clinical translation and provides a new strategy for efficient STING agonist delivery and immunotherapy of pediatric solid tumors.

## Graphical abstract

An engineered peptide self‐assembled nanofiber platform, designed to replicate STING–cGAMP binding motifs, facilitates pH‐responsive cytosolic delivery of cGAMP, enhances the endoplasmic reticulum with high efficiency, and robustly activates the STING pathway. This innovative system reprograms tumor‐associated macrophages towards an M1 phenotype and promotes the infiltration of CD8^+^ T‐cells, thereby effectively inhibiting neuroblastoma growth through both intratumoral and systemic administration.

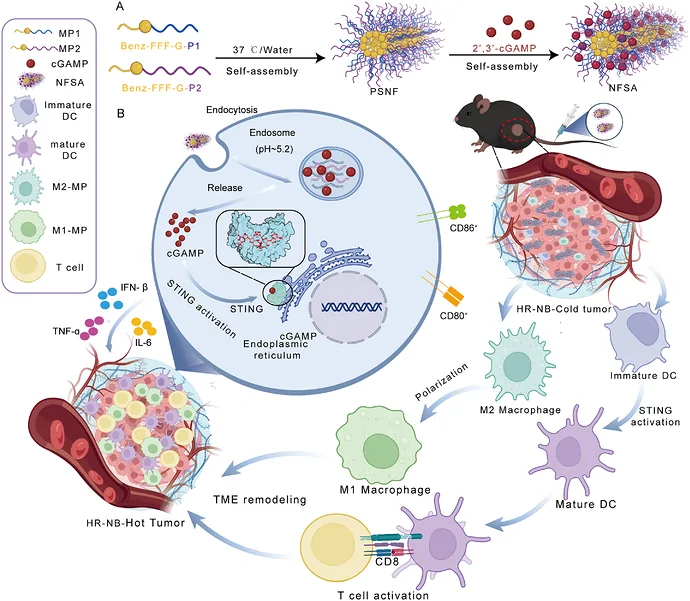

## 1 Introduction

Neuroblastoma (NB), a malignant pediatric solid tumor derived from neural crest cells, accounts for more than 15% of childhood cancer‐related mortality [1]. Despite intensive multimodal therapies, including surgical resection, stem cell transplantation, radiotherapy, chemotherapy, and anti‐GD2 monoclonal antibody immunotherapy, high‐risk patients have a 5‐year survival rate of less than 50%, with approximately 60% experiencing relapse and 20% progressing to therapy‐refractory disease states [2]. Chemotherapy resistance mediated by the tumor microenvironment (TME) further complicates clinical management. Recent advances in immunotherapy, such as immune checkpoint blockade (ICB), chimeric antigen receptor T‐cell (CAR‐T) therapy, oncolytic viruses, and bispecific antibodies, have revolutionized paradigms of cancer treatment [3]. However, their efficacy is strongly correlated with the degree of T cell infiltration in the tumor microenvironment, resulting in limited responses in “cold tumors” characterized by T‐cell exclusion [4].

Gainst this backdrop, the cyclic GMP‐AMP synthase‐stimulator of interferon genes (cGAS‐STING) pathway has emerged as a key player due to its intrinsic T cell‐independent immune activation mechanism [5]. Upon detecting cytosolic DNA (e.g., tumor‐derived or pathogenic DNA), this pathway triggers a cascade of events: cGAS synthesizes the second messenger 2',3'‐cyclic GMP‐AMP (cGAMP), which binds to and activates STING [6]. This activation induces the secretion of type I interferons (IFN‐I) and pro‐inflammatory cytokines, while recruiting innate immune cells (e.g., macrophages, natural killer cells) to infiltrate tumors, thereby reprogramming the immunosuppressive TME and initiating adaptive antitumor immunity [7, 8].

However, the clinical translation of free cGAMP has been hindered by multiple challenges, including inherent hydrophilicity, short plasma half‐life, poor tumor‐targeting ability, susceptibility to degradation by extracellular nucleases, and low intracellular delivery efficiency [9, 10]. Moreover, STING agonists exhibit a narrow therapeutic window [11] in which inadequate dosing compromises therapeutic efficacy, while systemic administration may induce cytokine‐mediated inflammatory responses [12]. To address these challenges, nanotechnology‐based drug delivery systems (NDDS) have emerged as a research hotspot due to their high drug‐loading capacity, precise targeting ability, and controllable release properties. Reported nanocarriers for cyclic dinucleotide (CDN) delivery include liposomes [13], nanoparticles [14], exosomes [15], and hydrogels [16]. These systems enhance drug accumulation in tumor tissues, facilitate cellular internalization, and protect payloads from enzymatic degradation during transit, thereby improving drug stability and targeting specificity. Critically, they enable spatiotemporal control over innate and adaptive immune activation, amplifying antitumor efficacy [17].

However, no current STING agonist delivery systems have been approved for clinical use, likely due to the following reasons. First, because of the ultra‐hydrophilicity of cGAMP, carriers exhibit extremely low encapsulation efficiency for it, and it is prone to burst release in vivo. This not only elevates the risk of systemic inflammation but also shortens the circulatory half‐life, as the drug is rapidly cleared via the reticuloendothelial system (RES) or through serum protein adsorption, thereby necessitating frequent dosing [18]. Second, limited lysosomal escape capacity prevents STING agonists from reaching the ER, while the anionic/hydrophilic nature of cGAMP hinders transmembrane transport, thereby collectively compromising STING pathway activation [19]. Finally, some synthetic materials (e.g., cationic liposomes) pose risks of hemolysis or chronic toxicity, and batch‐to‐batch physicochemical variations (e.g., fluctuations in size/zeta potential) arising from complex manufacturing processes further impede clinical consistency [20, 21].

Peptide‐based self‐assembled nanocarriers offer a promising alternative, leveraging exceptional biocompatibility, engineerable molecular architecture, and biomimetic binding mechanisms [22]. Rational design of electrostatic interactions, hydrogen bonding, and π–π stacking enables the formation of stable drug complexes that enhance targeting efficiency and prolong circulation time [23], potentially overcoming the inherent druggability limitations of cGAMP. Inspired by the high‐affinity interactions between cyclic dinucleotides (CDNs) and the STING binding pocket [24], we employed Benz‐FFF‐G to hydrophobically modify two CDN‐specific binding amino acid sequences from the STING protein [25], P1 (SYYIGY) and P2 (RAGIKDRVYS), developing a supramolecular assembly with cGAMP that structurally mimics the receptor‐ligand binding mechanism between STING protein and CDNs. Benz‐FFF‐G is composed of hydrophobic amino acids phenylalanine (F) and glycine (G)) that were conjugated at the N terminus to benzoic acid.

**SCHEME 1:**
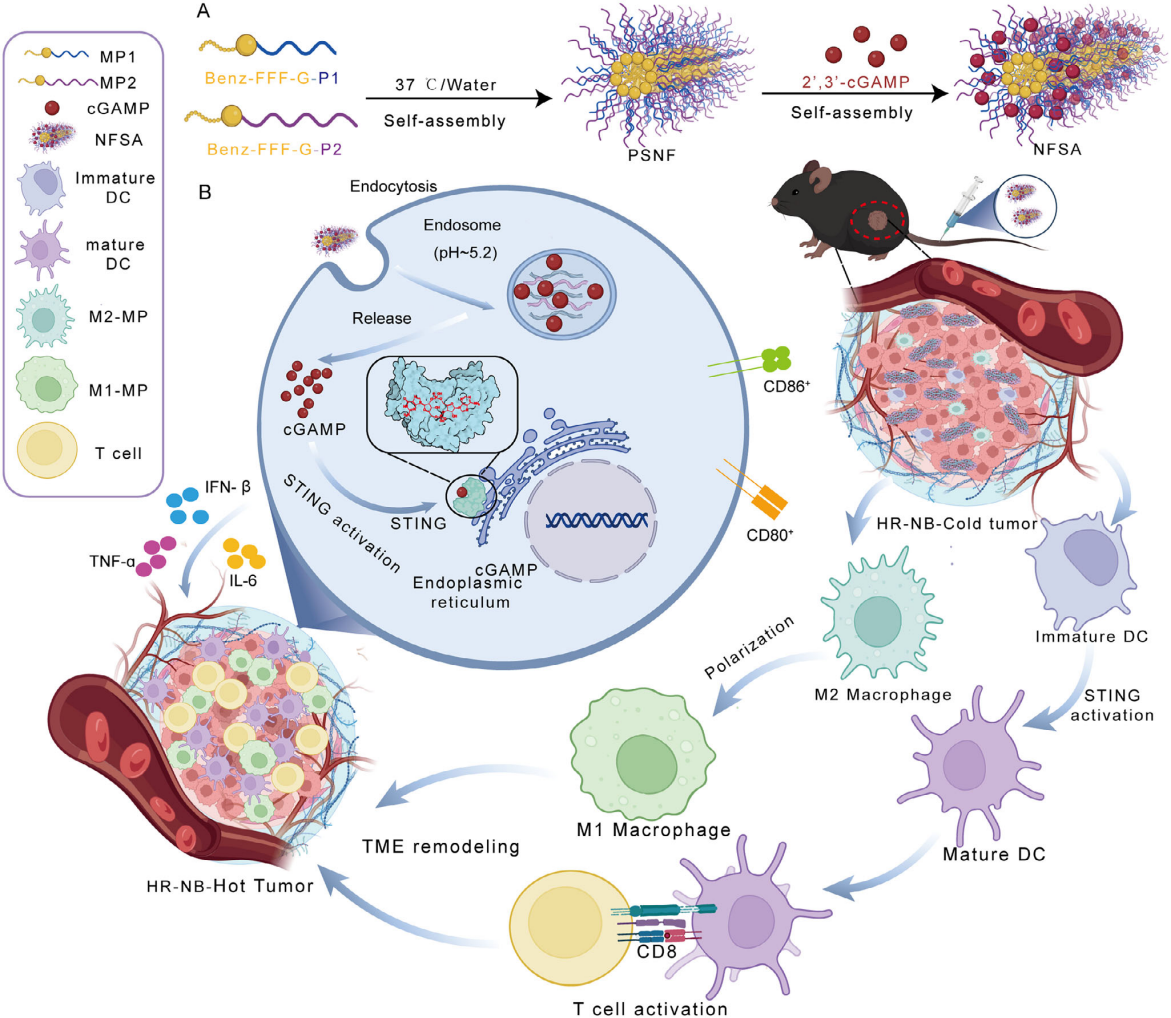
Schematic illustration of the construction of STING agonist‐loaded nanofibers (NFSA) and their application in cytosolic delivery of cGAMP for immunotherapy of mouse neuroblastoma. (A) Two amino acid sequences on the STING protein that specifically bind to CDNs, namely P1 (SYYIGY) and P2 (RAGIKDRVYS), are hydrophobically modified with Benz‐FFF‐G to obtain MP1 and MP2. These modified sequences assemble with cGAMP into nanofibers by structurally mimicking these receptor‐ligand interactions. (B) Upon cellular uptake, NFSA activates dendritic cells (DCs) via the STING pathway and triggers a robust antitumor immune response by repolarizing M2‐type macrophages to M1‐type macrophages. (Created with Biorender).

This STING agonist‐loaded nanofiber (NFSA) exhibits three key functional advantages. First, it mediates controlled drug release through balanced hydrophobic interactions and hydrogen bonding to minimize initial burst release while prolonging systemic circulation [26]. Second, the amphiphilic molecular structure designed in this study contains amino and guanidino groups, which may promote lysosomal escape of the nanofibers and enhance ER localization efficiency [27]. Third, the enhanced ER delivery enabled by this design has the potential to augment STING activation, thereby promoting the initiation of signaling cascades within the cGAS‐STING pathway [28]. To validate the immunotherapeutic potential of NFSA for neuroblastoma, we will first investigate the cytokine co‐expression patterns and secretion profiles in bone marrow‐derived dendritic cells and macrophages [29, 30]. Subsequently, its antitumor efficacy will be rigorously evaluated in high‐risk neuroblastoma mouse models via both intratumoral and systemic administration routes [31]. Finally, the prospects for clinical translation will be comprehensively analyzed through safety assessments and evaluations of the convenience of administration routes (Scheme 1).

## 2 Materials and Methods

### 2.1 Materials

2’,3’‐cGAMP (sodium) was purchased from MedChemExpress (MCE). All peptides were designed in‐house and synthesized by GiL Biochemistry via Fmoc solid‐phase peptide synthesis. Fluorescently conjugated anti‐mouse monoclonal antibodies (for flow cytometry) were obtained from Biolegend. Anti‐mouse monoclonal antibodies for immunofluorescence staining were obtained from Abcam and Biolegend. IL‐6 and TNF‐α enzyme‐linked immunosorbent assay (ELISA) kits were purchased from Biolegend. IP‐10, IFN‐β, IL‐4, and HMGB1 ELISA kits were purchased from Jianglai Biotechnology Co., Ltd. Murine DC 2.4, human neuroblastoma SH‐SY5Y, and human monocyte/macrophage THP‐1 cells were purchased from China Center for Type Culture Collection (CCTCC). 9464D neuroblastoma cells were obtained from Dr. Rimas Orentas at Seattle Children's Research Institute. RAW‐Blue ISG reporter cells and QUANTI‐Blue were purchased from InvivoGen. The wild‐type (WT) RAW 264.7 cells and STING1‐Knockout (KO) RAW 264.7 cells were purchased from Guangzhou Yuanjing Biotechnology Co., Ltd. Human STING1 Full‐Length Protein (Detergent) was purchased from Suzhou Perotin Biotechnology Co., Ltd. Cells were cultured at 37°C in a humidified atmosphere containing 5% CO_2_. Female C57BL/6 mice (18–20 g) were obtained from Beijing Charles River Laboratory Animal Technology Co., Ltd. All animal experiments were conducted in accordance with the guidelines of the Institutional Animal Care and Use Committee (IACUC) of Peking University Health Science Center and were approved by the Animal Ethics Committee of Peking University Health Science Center (Approval No. BCJE0149). All procedures were performed in accordance with the ARRIVE guidelines.

### 2.2 Peptide Sequence Selection and Synthesis

Inspired by the biomimetic principle of CDNs specifically binding to the STING protein, we designed peptide‐based carriers using the natural ligand cGAMP as the model drug. Two critical peptide sequences (P1: SYYIGY; P2: RAGIKDRVYS), corresponding to key binding sites on the STING protein, served as structural motifs. To facilitate self‐assembly of P1 and P2 and enhance their binding with cGAMP, hydrophobic Benz‐FFF‐G groups were introduced to modify these peptides into amphiphilic derivatives. This modification enabled the peptides to self‐assemble into nanoscale formulations in aqueous environments, thereby facilitating targeted drug delivery in vivo.

Molecular docking simulations were performed to validate binding affinity between hydrophobically modified peptides and cGAMP. Protonation states of the two modified peptides (MP1, MP2) were set at pH 7.4, and their 3D structures were generated using Open Babel. AutoDock Tools (ADT 3) was employed to prepare MP1, MP2, and cGAMP for docking procedures. The docking grid boxes were defined using AutoGrid, and subsequent molecular docking simulations were conducted utilizing AutoDock Vina (version 1.2.0). The optimal binding conformations were selected for interaction analysis, and PyMOL was used to generate interaction diagrams for the MP1‐cGAMP and MP2‐cGAMP complexes [32].

Finalized MP1 and MP2 sequences were synthesized by Gil Biochemical Co. The mass spectrometry analyzed relative molecular weights of the synthesized peptides to confirm sequence consistency with the intended targets.

### 2.3 Preparation and Screening of NFSA Formulations

MP1 (Figure S1), whose structure was verified via mass spectrometry, was accurately weighed and dissolved in dimethyl sulfoxide (DMSO). The solution was then dispersed in an aqueous medium. cGAMP (Figure S1) was incorporated into the peptide‐based nanocarrier solution at various molar ratios through ice‐water bath sonication (1 min) followed by vigorous stirring. The resultant mixtures were incubated overnight at 4°C to facilitate self‐assembly of the peptide‐drug formulation MP1SA. Using identical procedures, MP2 (Figure S1) was processed to generate the analogous formulation MP2SA.

**FIGURE 1:**
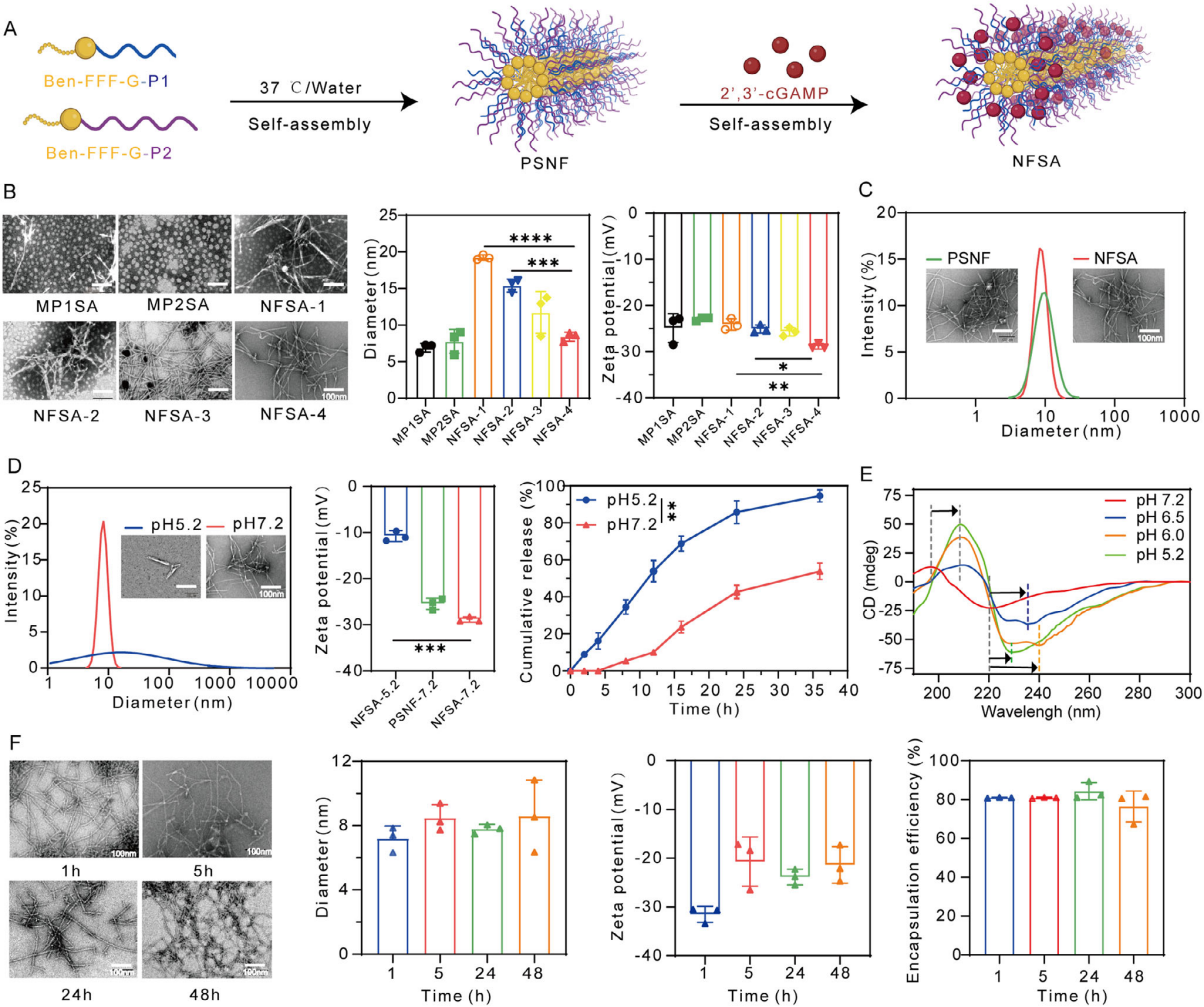
Physicochemical characterization of NFSA. (A) Preparation process of nanofibers loaded with STING agonist (NFSA). (B) Representative TEM images, particle sizes, and zeta potentials of peptide preparations formulated with varying ratios of MP1, MP2, and cGAMP. Specific preparation ratios are detailed in Figure S2. scale bar: 100 nm. (C) TEM images and particle size distribution of peptide formulations before and after drug loading. scale bar: 100 nm. (D) Particle size distribution, TEM images, zeta potential and drug release of NFSA at pH 7.2 and 5.2. scale bar: 100 nm. (E) Circular dichroism spectra of NFSA under different pH conditions. (F) TEM images, particle size, zeta potential and drug encapsulation efficiency of peptide formulations dispersed in PBS for 1, 5, 24, and 48 h. scale bar: 100 nm. *n* = 3. Data are presented as mean ± SD.*P < 0.05, **p < 0.01, ***p < 0.001, ****p < 0.0001.

Three combinatorial formulations (NFSA‐1, NFSA‐2, and NFSA‐3; Figure 1A; Table S1) were systematically fabricated by adjusting molar ratios of components to cGAMP:MP1:MP2 = 4:21:7, 4:20:4, and 4:20:5, respectively, while maintaining consistent preparation parameters. The optimal formulation was determined based on peptide solubility, electron microscopy analysis, particle size distribution, zeta potential, and encapsulation efficiency.

To ensure formulation safety, DMSO was removed, and NFSA‐3 was further optimized based on experimental results. The protocol was as follows: Peptides MP1 and MP2 were dissolved in DMSO, sonicated for 1 min, and dispersed in 2 mL aqueous solution. cGAMP was added to the peptide solution at a molar ratio of cGAMP:MP1:MP2 = 4:20:5, followed by thorough stirring and overnight incubation at 4°C. After ultrafiltration against pure water (three washes at 4000 rpm for 15 min each) to remove DMSO, the peptide self‐assembled nanomedicine NFSA‐4 (namely NFSA) was obtained (Figure 1A). The control formulation, peptide self‐assembled nanofibers (PSNF), was fabricated through the cooperative self‐assembly of MP1 and MP2 peptides without drug loading. Utilizing MP1’ (Benz‐FFF‐G‐YGYISY) and MP2’ (Benz‐FFF‐G‐RAGIKDRVYS) with identical charge densities but with randomized sequences, cGAMP was loaded employing the same preparation method, resulting in the acquisition of the control peptide chain (Ctrl‐NFSA).

### 2.4 Physicochemical Characterization of NFSA

The morphology of the resulting formulation was characterized by transmission electron microscopy (TEM) using 1.0% (w/w) uranyl acetate for negative staining. Hydrodynamic size and zeta potential of the nanoparticles were measured via dynamic light scattering (DLS) and Zetasizer Nano ZSP, respectively. Drug encapsulation efficiency was determined by high‐performance liquid chromatography (HPLC).

### 2.5 Stability and pH Sensitivity of the Optimized NFSA Formulation

Following the preliminary physicochemical characterization described above, NFSA‐4 was selected as the optimized formulation for subsequent investigations and hereafter referred to as NFSA. For stability assessment, freshly prepared NFSA nanoparticles were dispersed in phosphate‐buffered saline (PBS) and 10% fetal bovine serum (FBS), and temporal changes in morphology, hydrodynamic diameter, zeta potential, and encapsulation efficiency were systematically monitored at predefined time intervals.

To evaluate pH responsiveness, NFSA dispersions were prepared in PBS adjusted to physiological (pH 7.2) and acidic (pH 6.5 and pH 5.2) conditions, mimicking normal tissue and tumor microenvironments, respectively. Morphological analysis under varying pH was performed using transmission electron microscopy (TEM) with 1.0% (w/w) uranyl acetate negative staining. Hydrodynamic diameter and surface charge were determined via dynamic light scattering (DLS) and electrophoretic light scattering (ELS) using a Zetasizer Nano ZSP. The secondary structure of NFSA was examined at pH 7.2, 6.5, 6.0, and 5.2 using circular dichroism (CD) spectroscopy at a temperature of 25°C in a phosphate buffer solution. Spectral data were recorded across wavelengths from 190 to 300 nm.

In vitro drug release profiles were investigated using an ultrafiltration‐based method. Briefly, triplicate samples of free cGAMP‐Cy5 and NFSA‐Cy5 (the cyanine‐5 (Cy5)‐labeled cGAMP was encapsulated within self‐assembling peptide nanofiber carriers) were incubated in pH 7.2, pH 6.5, and pH 5.2 PBS at 37°C with gentle agitation (100 rpm). At designated time points (0.5, 1, 2, 4, 6, 8, 10, 16, 24, 36, and 48 h), ultrafiltration tubes were centrifuged, and both filtrate and retentate fractions were collected. Fluorescence intensity was measured using a spectrofluorometer, and cumulative drug release was calculated using pre‐established calibration curves. Release kinetics were visualized by plotting cumulative release percentage against time.

### 2.6 Cellular Uptake and Distribution

Logarithmically growing RAW 264.7, 9464D, and DC 2.4 cells were seeded into 24‐well plates and cultured overnight. Cells were then treated with free cGAMP‐Cy5 or NFSA‐Cy5 at equivalent cGAMP‐Cy5 concentrations for 2, 4, or 8 h (*n* = 3). Following incubation, cells were harvested and analyzed by flow cytometry to quantify intracellular fluorescence intensity. Confocal laser scanning microscopy (CLSM) was employed for qualitative assessment of cellular uptake and subcellular localization.

To investigate the co‐localization of the ER, DC 2.4 cells were seeded at a density of 2500 cells per well in a confocal culture dish and incubated overnight to allow for adequate growth. Subsequently, the cells were exposed to free cGAMP‐Cy5, NFSA‐Cy5, and Ctrl‐NFSA‐Cy5, each at a concentration of 5 µg mL^−1^ cGAMP‐Cy5, for durations of 0.5, 2, and 4 h. The nuclei and ER were stained with DAPI and ER‐Tracker Green, respectively, for 15 min. Following the removal of the supernatant, Lyso‐tracker was added to facilitate the observation of cGAMP‐Cy5 co‐localization with lysosomes and the ER using CLSM. Quantitative co‐localization analysis was conducted utilizing Plot Profile and Pearson correlation coefficient in ImageJ software.

The RAW 264.7 cells, exhibiting logarithmic growth, were seeded into 24‐well plates and cultured overnight. Subsequently, three endocytosis inhibitors—chlorpromazine (CPZ, 10 µg mL^−1^), amiloride (AMI, 0.1 mm), and genistein (GEN, 0.1 mm)—were administered for one hour each. This was followed by treatment with equivalent concentrations of cGAMP‐Cy5, NFSA‐Cy5, and Ctrl‐NFSA‐Cy5 for durations of 2, 4, or 8 h (*n* = 3). Post‐incubation, the cells were harvested, and their intracellular fluorescence intensity was quantified via flow cytometry. The inhibition rate was then calculated.

### 2.7 STING Dependency Verification Experiments

STING pathway activation was evaluated using RAW‐Blue ISG reporter cells. Logarithmically growing cells were seeded into 96‐well plates at 4–5×10^4^ cells per well and cultured overnight. Varying concentrations of free cGAMP, PSNF, and NFSA were added to DMEM medium, followed by incubation at 37°C under 5% CO_2_ for 24 h. Post‐incubation, 50 µL of supernatant was mixed with 150 µL of QUANTI‐Blue reagent and incubated at 37°C with gentle shaking until color development. Absorbance was measured at 620 nm [33].

RAW 264.7 cells and STING1 knockout cells, both in the logarithmic phase of growth, were seeded into 6‐well plates and cultured overnight. Subsequently, the cells were exposed to equal concentrations of cGAMP, PSNF, and NFSA for a duration of 8 h (*n* = 3). Following incubation, supernatants were collected for the quantification of cytokines IP‐10 and IFN‐β using ELISA kits. Concurrently, cells were harvested for the analysis of phosphorylated proteins associated with the STING signaling pathway, which was conducted via Western blotting.

SH‐SY5Y, 9464D, and THP‐1 cells in the logarithmic growth phase were seeded into 6‐well plates and cultured overnight. They were then treated with NFSA for various durations (0, 0.5, 1, 1.5, 2, 3, and 5 h, *n* = 2 per time point), with corresponding blank controls. Post‐incubation, supernatants were collected to measure HMGB1 levels via ELISA, proteins were extracted to assess STING pathway phosphorylation (p‐TBK1, p‐IRF3, p‐STING) using Western blot, and ATP levels were measured using bioluminescence.

### 2.8 Surface Plasmon Resonance Experiment

The experiment used the Biacore 8K system with a CM5 sensor chip, immobilizing human STING1 on the sample channel (Fc2) at about 12 600 RU, while the reference channel (Fc1) served as a control. NFSA was diluted into 11 concentrations (0.78–50.00 µm) in a running buffer and injected at 20 µL min^−1^ with 60 s for binding and 90 s for dissociation. This process was repeated for seven cycles, with chip regeneration using 10 mm glycine‐HCl (pH 2.0) after each cycle. The experiment determined the dissociation constant (*K*_d_), association rate constant (*K*_a_), and dissociation rate constant (*K*_d_).

### 2.9 In Vitro Activation of BMDCs

Bone marrow‐derived dendritic cells (BMDCs) were isolated from the femurs and tibias of female C57BL/6 mice (18–20 g). Bones were repeatedly flushed with PBS until debris‐free, and the bone marrow suspension was filtered through a 70‐µm cell strainer. Cells were seeded in culture dishes and supplemented with 10 ng mL^−1^ recombinant murine interleukin‐4 (rmIL‐4) and 25 ng mL^−1^ recombinant murine granulocyte‐macrophage colony‐stimulating factor (rmGM‐CSF) to promote maturation. Half of the culture medium was replaced on days 2, 4, and 6. On day 7, cells were transferred to 24‐well plates for treatment.

BMDCs were incubated with PBS (control), free cGAMP, PSNF, or NFSA at an equivalent cGAMP dose of 10 µg mL^−1^. After 24 h, cells were harvested, blocked with anti‐mouse CD16/32 antibody for 15 min on ice, and stained with fluorochrome‐conjugated antibodies against CD11c (FITC), CD80 (PE/Cy7), and CD86 (APC) (Biolegend) for 30 min on ice. Following centrifugation, cells were washed with cell staining buffer and resuspended in 500 µL of PBS. The surface expression levels of CD80 and CD86 were analyzed using flow cytometry. Supernatants were collected, and cytokine levels (IL‐6, IFN‐β, and TNF‐α) were quantified using ELISA kits (Biolegend) according to the manufacturer's instructions.

### 2.10 In Vitro Polarization Phenotype of BMDMs and THP‐1 Cells

Bone marrow‐derived macrophages (BMDMs) were extracted from the femurs and tibias of female C57BL/6 mice, weighing between 18 and 20 g. These cells were cultured in heat‐inactivated DMEM medium supplemented with 20 ng mL^−1^ recombinant mouse macrophage colony‐stimulating factor (rmM‐CSF) for a duration of five days, resulting in the generation of M0‐like BMDMs. THP‐1 cells were differentiated into M0‐like THP‐1 macrophages through induction with 100 nm phorbol ester. To further differentiate these cells into M1 and M2 macrophage phenotypes, the M0‐like BMDMs and M0‐like THP‐1 cells were exposed to 100 ng mL^−1^ lipopolysaccharide (LPS) in conjunction with 20 ng mL^−1^ recombinant mouse interferon‐γ (rmIFN‐γ) or 20 ng mL^−1^ recombinant mouse interleukin‐4 (rmIL‐4) for 24 and 48 h, respectively. Following polarization, the macrophages were subjected to additional treatment with either PBS, cGAMP, PSNF, or NFSA for 24 h.

Harvest the cells and stain them with macrophage markers, specifically mouse‐derived CD11b and human‐derived CD68, as well as M1‐type marker CD86 and M2‐type marker CD206, utilizing fluorescent dyes for a duration of 30 min. Subsequently, wash the cells with a cell staining buffer, centrifuge them, and resuspend in 200 µL of PBS. Employ flow cytometry to analyze the proportion of CD206^+^ and CD86^+^ macrophages. Collect the cell culture supernatant and quantify the concentrations of IL‐6, IL‐4, TNF‐α, IFN‐β, and Arg‐1 using ELISA kits, following the manufacturer's protocols. Determine the nitrite concentration via the Griess colorimetric method, serving as an alternative measure of nitric oxide (NO) production. Additionally, evaluate the expression of arginase‐1 (Arg‐1) protein within the cells through Western blot analysis, utilizing the total protein lysate from the treated cells.

### 2.11 Therapeutic Efficacy and Immune Activation of Intratumoral NFSA Administration

Female C57BL/6 mice (18–20 g) were subcutaneously inoculated with 1 × 10^6^ 9464D cells in 50 µL PBS on the right flank. For therapeutic efficacy assessment, mice were randomized on day 9 post‐inoculation into six groups (*n* = 8 per group) receiving intratumoral injections of PBS, PSNF, free cGAMP, or NFSA on days 10, 13, and 16 (5 µg cGAMP equivalent per mouse). Tumor volumes were calculated using *V* = (*a*×*b* ^2^)/2. Mice were euthanized at 1500 mm^3^ for survival analysis. Tumor tissues were processed for multiplex immunohistochemistry using anti‐CD8^+^, CD4^+^, iNOS^+^, CD206^+^ antibodies (Abcam), amplified with Wellgene kits and analyzed via Vectra Polaris system.

For immune activation and safety evaluation, mice were randomized on day 10 post‐inoculation (*n* = 8 per group) for intratumoral treatments with PBS, PSNF, free cGAMP, or NFSA (5 µg cGAMP per mouse). Tumor dimensions and body weight were measured every two days. Blood samples were obtained via ocular enucleation and exsanguination in mice for biochemical analysis (hepatorenal function) and ELISA (IL‐6, IFN‐β, TNF‐α), H&E staining of tumors and major organs (heart, liver, spleen, lung, and kidney), and flow cytometry of immune markers (CD4^+^, CD8^+^, CD80^+^, CD86^+^) in tumor‐draining lymph nodes and tumor homogenates.

### 2.12 Antitumor Preventive Effects, Antitumor Efficacy, In Vivo Evaluation of Tumor‐Targeted Delivery and Immune Activation by Systemic Nanomedicines

To evaluate the preventive effect of NFSA on high‐risk neuroblastoma, female mice weighing 18–20 g were randomly divided into 4 four groups (*n* = 6). The animals were administered with PBS, PSNF, cGAMP, or NFSA by tail vein injection (cGAMP 5 µg per mouse) on day ‐9, day ‐6, and day ‐3. On day 0, mice were subcutaneously inoculated with 2 × 10^6^ tumor cells per mouse. For the neuroblastoma tumor model, 9464D cells were inoculated in the right buttock of mice. The long (*a*) and short (*b*) diameters of the tumors were measured every 2 d using vernier calipers. Tumor volume was calculated according to the formula: Tumor volume = (*a* × *b* ^2^)/2, and the mice were weighed. A tumor volume up to 1500 mm^3^ was used as the endpoint of the experiment, and the survival period of the mice was recorded.

For the murine neuroblastoma animal model, female C57BL/6 mice (18–20 g) were subcutaneously implanted with 1 × 10^6^ 9464D cells in 50 µL PBS into the right flank. For biodistribution analysis, mice with 150–250 mm^3^ tumors were randomized (*n* = 3 per group) and intravenously injected with Cy5‐labeled formulations. In vivo fluorescence imaging was performed at 0.5‐72 h post‐injection using a PerkinElmer IVIS Lumina system and analyzed with Living Image software (Caliper Life Sciences).

For efficacy and immune activation studies, mice were randomized on day 11 (efficacy) or 17 (immune) post‐inoculation into treatment groups (*n* = 8 per group) and treated with PBS, PSNF, free cGAMP, or NFSA (5 µg cGAMP per mouse) via tail vein injection on days 12, 15, 18 (efficacy) or 17, 20, 23 (immune). Tumor volumes were calculated using *V* = (*a* × *b* ^2^)/2. Mice were euthanized at 1500 mm^3^ for survival analysis. Tumor‐draining lymph nodes were processed for multiplex immunohistochemistry (anti‐CD8^+^/CD4^+^, Abcam), analyzed via Wellgene kits and Vectra Polaris system (PerkinElmer). Blood collected via retro‐orbital puncture post‐treatment was analyzed for serum biochemistry and ELISA (IL‐6, TNF‐α). Necropsy specimens were formalin‐fixed for H&E staining and flow cytometry (CD4^+^, CD8^+^, CD80^+^, CD86^+^ immune cells). Body weight was monitored throughout the entire experiment.

### 2.13 Proteomic Analysis of the Protein Corona

Serum from C57BL/6 mice was incubated with NFSA to mimic physiological conditions for protein corona formation, followed by agitation at 220 revolutions per minute (rpm) at 37°C for 1 h. After incubation, centrifugation at 15 000 × *g* for 30 min was performed to pellet NFSA‐P, defined as NFSA particles coated with the protein corona. Unbound proteins were removed via three consecutive washing steps with ice‐cold phosphate‐buffered saline (PBS).

The total protein amount of the harvested protein corona was quantified using a bicinchoninic acid (BCA) protein assay kit (Thermo Fisher Scientific, USA). Proteins eluted from the NFSA‐P surface were subjected to reduction, alkylation, and tryptic digestion to prepare samples for mass spectrometry (MS) analysis. The final peptide samples were analyzed using a Thermo Scientific Orbitrap Astral mass spectrometer to identify and profile the protein composition of the corona. Identified proteins were ranked by relative abundance, and their theoretical isoelectric points (pI) were calculated using the ExPASy bioinformatics resource. The identified proteins were subsequently classified into functional categories based on annotations retrieved from the UniProtKB database.

### 2.14 Immune Serological Verification

Antibody titers in mouse serum samples were determined by ELISA. MP1 and MP2 were each diluted to 5 µg mL^−1^ with 0.05 m carbonate coating buffer (pH 9.6). A total of 100 µL of the diluted solution was added per well of 96‐well microplates, followed by overnight incubation at 4°C. Subsequently, the wells were washed three times with phosphate‐buffered saline containing 0.05% Tween‐20 (PBST) and then blocked with blocking buffer.

Mouse serum from each group, diluted at 1:100 (v/v) as the primary antibody, was then added and incubated. After washing, the secondary antibody was applied and incubated, followed by the addition of 3,3',5,5'‐tetramethylbenzidine (TMB) substrate for chromogenic reaction. Finally, the reaction was terminated by adding stop solution, and the optical density (OD) value was measured at 450 nm using a microplate reader.

The negative control used in this study consisted of serum from non‐immunized C57BL/6 mice, pooled from ten mice. The positive control consisted of high‐titer immune serum specific to MP1/MP2, while the blank control was set as diluent devoid of serum.

For preparation of positive serum, Cys‐MP1 or Cys‐MP2 peptides were conjugated to the carrier protein bovine serum albumin (BSA), subsequently emulsified with Freund's adjuvant, and administered to C57BL/6 mice via intraperitoneal injection and multi‐site subcutaneous injection (25 µg/100 µL, totaling 100 µg per mouse). Two booster immunizations were performed on days 14 and 28 after the primary immunization, with serum samples collected on day 35 for titer determination. The positive cutoff value was determined as the mean OD value of the negative serum (*n* = 6) plus two times the standard deviation (SD).

### 2.15 Biosafety and Toxicity Assessments of NFSA Formulation

#### 2.15.1 Cellular Toxicity Evaluation (CCK‐8 Assay)

RAW264.7 macrophages in the logarithmic growth phase were seeded into 96‐well plates and incubated overnight. Cells were treated with PBS, free cGAMP, PSNF, or NFSA at various cGAMP concentration gradients (30, 15, 7.5, 3.75, 1.875, 0.9375 µm; *n* = 3 percondition) for 24 h at 37°C with 5% CO_2_. Post‐incubation, 10 µL of CCK‐8 reagent was added to each well. The plates were incubated for 0.5–4 h, and absorbance was measured at 450 nm to determine cell viability.

#### 2.15.2 In Vitro Hemocompatibility

Red blood cell (RBC) suspensions were incubated with 200 µL of test formulations (NFSA, free cGAMP, PSNF) at 37°C. Following centrifugation, hemoglobin release was quantified by absorbance at 540 nm. Negative (PBS) and positive (Triton X‐100) controls were included. Hemolysis rate (%) was calculated as:

$$Hemolysis\;rate=\frac{Abs_{sample}-Abs_{PBS}}{Abs_{Rriton\;X-100}-Abs_{PBS}}\times 100\%$$

where Abs_sample_, Abs_negative_, and Abs_positive_ denote the absorbance values of the NFSA, PBS, and Triton X‐100 groups, respectively.

#### 2.15.3 In Vivo Toxicity and Histopathological Analysis

Mice were weighed every other day during treatment. At study endpoints, tissues (heart, liver, spleen, lung, kidney) from three mice per group were fixed in 4% paraformaldehyde, paraffin‐embedded, and sectioned at 4 µm for H&E staining. Serum biochemical markers (ALT, AST, CREA, BUN, UA) were analyzed via ocular blood collection to assess hepatorenal function.

### 2.16 Statistical Analysis

Data were presented as mean ± standard deviation (s.d.) or mean ± standard error of the mean (s.e.m.), analyzed with GraphPad Prism 8 and OriginPro 9.1. The significance of the differences among groups was analyzed by student's t‐tests or ANOVA followed by Tukey's multiple comparisons test for post hoc tests. *p < 0.05, **p < 0.01, ***p < 0.001, ****p < 0.0001.

## 3 Results and Discussion

### 3.1 Molecular Design and Characterization of Peptide‐Based Self‐Assembled Nanocarriers

The specific, high‐affinity interaction between cGAMP and the STING protein forms the basis for our nanocarrier design. As illustrated in Figure S2A,B, cGAMP is deeply embedded within the binding pocket of the STING dimer, engaging multiple amino acid residues through specific molecular interactions: The purine base of cGAMP exhibits π‐π stacking with aromatic residues Tyr240 and Tyr167 from both STING monomers. Its two α‐phosphate groups form electrostatic interactions with Arg238 and Arg232 of the STING subunit, while the free 3'‐OH group is oriented toward two Ser162 residues at the base of the pocket. Key β‐sheet residues (Arg232, Arg238, Val239) involved in STING binding may regulate the transition to a closed conformation [34]. Leveraging this structural insight, we designed two peptide sequences (P1: SYYIGY; P2: RAGIKDRVYS) containing these critical amino acid residues and modified their hydrophobic domains to create peptide‐based nanomaterials (Modified Peptides, MPs: MP1 and MP2, denoting modified P1 and P2, respectively). These MPs were engineered to form a sustained‐release self‐assembly system through specific binding with CDNs. Previous studies have shown that non‐covalent interactions (hydrogen bonding, electrostatic forces, hydrophobic effects, π–π stacking, and van der Waals forces) collectively dictate the structural and functional properties of peptide self‐assemblies [23, 35], with hydrophobic interactions playing a primary role in driving amphiphilic molecular organization—crucially modulating drug‐peptide distance and interaction strength [36]. Given cGAMP's aromatic moieties and the enhanced stability conferred by aromatic‐aromatic interactions, we incorporated the established self‐assembling hydrophobic motif FFF (trifluorophenyl) [37]. To optimize the linkage between the FFF tail and the P1/P2 sequences, glycine (G) was selected as a flexible linker due to its small size and conformational adaptability [38]. Additionally, benzoic acid (Benz) was introduced at the N‐terminal of FFF to provide hydrogen bond donor/acceptor capabilities through its carboxyl group, facilitating the formation of strong centrosymmetric hydrogen‐bonded dimers [39]. This design resulted in the Benz‐FFF‐G hydrophobic modification, which balances the hydrophilic‐hydrophobic ratio and regulates peptide‐drug binding efficiency.

Molecular docking simulations (Figure S2C) revealed a binding free energy of −5.8 kcal mol^−1^ for the MP1‐cGAMP complex, primarily mediated by a 2.9 Å hydrogen bond between the phosphate group of Tyr5 and cGAMP. MP2 exhibited a binding energy of −4.5 kcal mol^−1^ through hydrogen bond interactions with Arg (3.4 Å) and Lys (3.3 Å). Both theoretical calculations and experimental data confirm that MP1/MP2, modified with hydrophobic domains, achieve specific cGAMP binding through multiple non‐covalent interactions [40].

Synthesized MPs were characterized by mass spectrometry, yielding molecular weights of 1367.54 (MP1) and 1767.04 (MP2) (Figure S2D,E). Self‐assembly with cGAMP was evaluated via TEM and DLS. MP1 and MP2 alone formed nanoparticles (Figure 1A) with diameters of 6.91 ± 0.58 nm (MP1SA) and 7.78 ± 1.62 nm (MP2SA), exhibiting negative zeta potentials of −24.9 ± 3.12 and −22.93 ± 0.40 mV, respectively (Figure 1B).

Formulation screening across varying molar ratios (Table S1; Figure S3A) identified NFSA‐3 with the cGAMP:MP1:MP2 ratio of 4:21:5 as optimal, forming homogeneous nanofibers with a diameter of 11.72 ± 2.86 nm, low polydispersity of 0.052 ± 0.008, and high encapsulation efficiency of 74.33% (Table S1). Ultrafiltration further improved EE to 81.09% (NFSA‐4), with preserved nanofiber morphology and reduced size (8.99 ± 0.23 nm). Drug loading (MP1:MP2:cGAMP = 20:5:4) induced structural compaction (8.02 ± 0.18 nm) and increased surface charge (‐28.7 ± 1.2 mV), suggesting electrostatic stabilization (Figure 1B,D).

To investigate the impact of cGAMP loading on nanostructures, composite systems with a molar ratio of MP1:MP2:cGAMP = 20:5:4 were prepared. TEM observations showed that the fibrous morphology persisted after drug loading (Figure 1C, but the particle size significantly decreased to 8.02 ± 0.18 nm (*p* < 0.05) with a reduced PDI (0.042 ± 0.005). The absolute zeta potential increased from −25.47 ± 1.22 to −28.7 ± 1.2 mV (Figure 1B,D), suggesting that drug loading may modulate surface charge distribution through electrostatic interactions.

Under acidic conditions of pH 5.2, TEM revealed short nanofibers and irregular aggregated nanofibers, with a particle size of 17.07 ± 0.94 nm and a zeta potential of −10.79 ± 1.19 mV. In contrast, at physiological pH 7.2, the nanofibers exhibited stable structural integrity, uniform distribution, and a reduced particle size (8.02 ± 0.15 nm) accompanied by a more negative zeta potential (−28.87 ± 0.57 mV) (Figure 1D; Figure S3B). In vitro release studies Figure 1D) demonstrated pH‐responsive drug release behavior. Under conditions characterized by a pH of 5.2, which is indicative of an acidic environment., approximately 16% of the drug was rapidly released within the initial 4 h, potentially due to the dissociation of surface‐adsorbed drug molecules, followed by sustained release reaching about 82% over 24 h. Conversely, under physiological conditions (pH 7.2), only 45% cumulative release was observed within 24 h, with significantly slower release kinetics compared to the acidic environment.

The release kinetics and size alterations of the nanomedicine were assessed under pH 6.5 conditions to emulate the tumor microenvironment. As illustrated in Figure S4A,B, the system demonstrated a continuous biphasic release profile, achieving a cumulative release of 51.8% over 48 h. Furthermore, dynamic light scattering analyses (Figure S4C) revealed that the nanoparticles retained a relatively small initial size (approximately 60 nm) during the first 2 h, which is favorable for tumor‐targeted aggregation facilitated by the enhanced permeability and retention (EPR) effect. Importantly, after 4 h, the particle size markedly increased to 603.9 ± 99.77 nm, coinciding with the commencement of substantial drug release. This pH‐triggered swelling or dissociation behavior not only enhances the sustained release of therapeutic agents but may also promote cellular uptake by modifying the interaction between the particles and the cell membrane. These findings confirm the pH‐sensitive characteristics of the system, indicating that our formulation could effectively minimize off‐target effects in normal tissues while demonstrating tumor‐specific release of active pharmaceutical agents, thereby maximizing therapeutic efficacy in tumors.

Circular dichroism (CD) spectra of the peptide nanofibers were recorded at pH levels of 7.2, 6.5, 6.0, and 5.2 to investigate alterations in secondary structure in response to acidification (Figure 1E). At pH 7.2, the spectra displayed characteristic double minima at 208 and 222 nm, indicative of a predominantly α‐helical conformation. As the pH was decreased to 6.5, there was a notable reduction in negative ellipticity at both 208 and 222 nm, suggesting partial unfolding of the helical structure. At pH 6.0, the minimum at 208 nm was nearly absent, while the signal at 222 nm became more negative, indicating a transition towards β‐sheet/β‐turn conformations. Further acidification to pH 5.2 resulted in a single intense negative band spanning the entire wavelength range of 200–260 nm, a characteristic feature of random coil or aggregated states with a substantial loss of ordered secondary structure. These pH‐dependent conformational transitions—from α‐helix to β‐structure and ultimately to disordered aggregates—correlate with the destabilization of the fibrous architecture, potentially facilitating the release of encapsulated small‐molecule drugs by disrupting hydrophobic packing and electrostatic interactions within the nanofiber matrix.

The stability of the engineered nanosystem was first evaluated via a 72 h incubation test in PBS buffer (Figure 1F; Figure S4D). The nanoscaffold well retained its intact nanofibrous architecture and consistent particle size throughout the incubation period. Correspondingly, the zeta potential was stably maintained at −27.5 ± 2.1 mV, and the drug encapsulation efficiency plateaued at 72.4% ± 3.8%. Compared with previously reported liposomal CDN delivery vectors [41], this supramolecular nanosystem achieved a markedly enhanced drug encapsulation capability. This superiority can be primarily attributed to the multi‐site anchoring interactions between the supramolecular network and hydrophilic cGAMP molecules, which effectively immobilize payloads and reduce drug leakage. Such robust PBS stability over 72 h satisfies the fundamental solution‐state administration requirements for subsequent in vitro cellular assays and in vivo animal investigations.

To more accurately replicate the in vivo blood circulation environment, we assessed the variations in particle size and zeta potential of the NFSA over a 72‐hour period within a medium containing 10% FBS, as depicted in Figure S5A,B. The findings revealed that during the initial 0–8 h, there were no significant alterations in the particle size and charge of the delivery system, which remained at 8.75 ± 5.35 nm and −22.28 ± 4.64 mV, respectively. This observation suggests that the delivery system exhibits substantial stability in the early phase, without evidence of aggregation or dissociation, rendering it suitable for intravenous administration. Conversely, beyond the 8‐hour mark, a notable increase in particle size and a reduction in the absolute value of the charge were observed. This instability in the later stages was corroborated by Coomassie Brilliant Blue staining (Figure S5C), which indicated that protein adsorption‐induced aggregation was responsible for the observed changes.

### 3.2 NFSA Enhances Cytosolic Delivery of cGAMP and STING Activation

We then evaluated its cellular uptake via flow cytometry and confocal microscopy, STING pathway activation efficiency.

**FIGURE 2:**
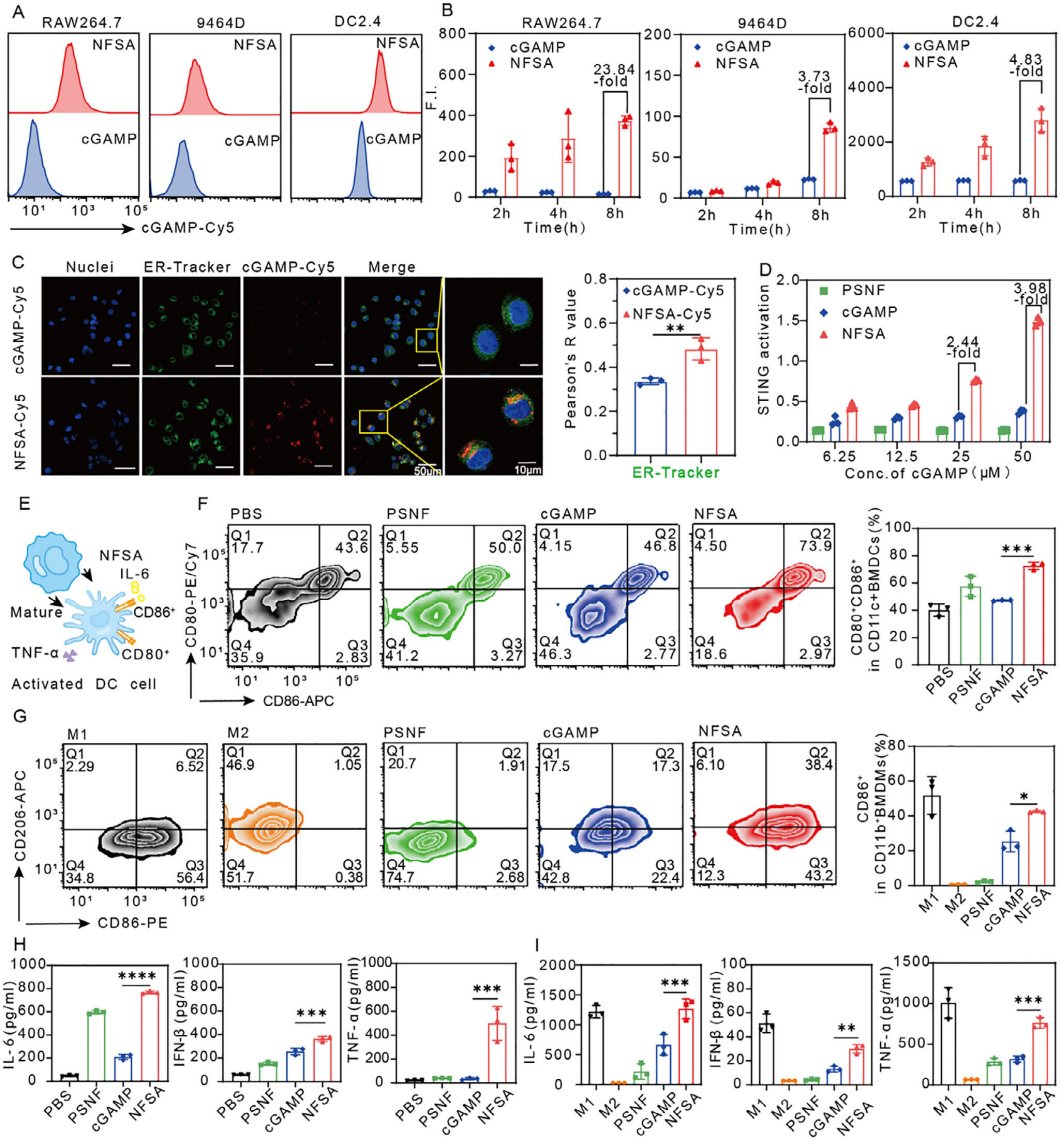
In vitro uptake and immunostimulation. (A, B) Flow‐cytometric quantification of Cy5‐labeled cGAMP uptake by RAW264.7, 9464D and DC 2.4 cells after 2, 4, and 8 h. *n* = 3. (C) Representative confocal images and Pearson correlation coefficients of ER co‐localization after 4 h incubation (scale bar, 50 µm). (D) Concentration‐dependent activation of the STING pathway by PSNF, free cGAMP, and NFSA, as determined by SEAP activity in Raw_Blue ISG cells. *n* = 3. (E) Schematic illustration of BMDC activation. (F) Flow cytometric analysis of BMDC maturation (CD80^+^CD86^+^ expression). *n* = 3. (G) Flow cytometric analysis of BMDM polarization (CD86^+^CD206^+^ BMDM phenotypes). (H) IL‐6, IFN‐β and TNF‐α secretion in treated BMDCs, and (I) in BMDMs, measured by ELISA (24 h, *n* = 3). Data are mean ± SD; *p < 0.05, **p < 0.01, ***p < 0.001, ****p < 0.0001.

Flow‐cytometry quantification showed that free cGAMP uptake was minimal in RAW264.7, 9464D and DC 2.4 cells, whereas NFSA increased uptake 25.8‐fold in RAW264.7 cells after 8 h (*p* < 0.0001, Figure 2A,B; Figure S6). Confocal imaging revealed time‐dependent ER accumulation of NFSA in DC 2.4 cells; the ER‐tracker colocalization coefficient reached 0.48 ± 0.05 at 8 h, versus 0.33 ± 0.01 for free cGAMP (*p* = 0.0085, Figure 2C, Figure S8A,B). The data corroborate that NFSA facilitates enhanced ER delivery, likely due to the guanidine side chains of MP2. These side chains exhibit a high affinity for phosphate backbones and demonstrate strong penetration capabilities through phospholipid bilayers, thereby conferring NFSA with a potent ability for endosomal escape [27].

To verify that the observed property promoting ER localization stems from the biomimetic design rather than from non‐specific charge‐mediated cellular uptake, we developed a control nanodelivery system as Ctrl‐NFSA (Figure S7A,B) that possess a similar charge density but feature randomly scrambled sequences. As depicted in Figure S7C–E, the particle size of Ctrl‐NFSA is 53.55 ± 4.78 nm, and its charge density aligns with that of NFSA. The morphology of Ctrl‐NFSA is characterized by an irregular nanofibrous structure. The findings obtained from CLSM and Plot Profile images (Figure S8C) indicate that the uptake of the control nano‐formulation, Ctrl‐NFSA‐Cy5, progressively increased over time, predominantly co‐localizing with lysosomes and exhibiting no significant co‐localization with the ER. Conversely, the organized assembly of biomimetic peptides in NFSA seems to promote an energy‐dependent internalization pathway that partially circumvents lysosomal entrapment. In contrast, randomization of the peptide sequence disrupts this structural organization, redirecting the nanoparticles towards lysosomal degradation. These findings indicate that the structural integrity of the biomimetic peptide scaffold, rather than merely charge effects, is crucial in determining the intracellular trafficking pathway.

Subsequently, we conducted an in‐depth investigation into the endocytic pathway utilized by NFSA to penetrate cells. Prior to the incubation of NFSA with RAW264.7 cells, the cells were pre‐treated for 1 h with three distinct endocytosis inhibitors: AMI, CPZ, and GEN. As demonstrated in Figure S9, flow cytometry analysis indicated that 8 h post‐administration, the inhibition rates of AMI, CPZ, and GEN on cells treated with NFSA were 33.54%, 43.85%, and 51.18%, respectively. Notably, GEN exhibited the most significant inhibitory effect, with an inhibition rate surpassing 50%. These results suggest that NFSA primarily employs the caveolae‐mediated endocytosis pathway for cellular entry, which may facilitate lysosomal evasion and subsequently extend its therapeutic effects.

Using the RAW‐Blue‐ISG cells to assess STING pathway activation, we found that NFSA containing 25 µm cGAMP induced 2.44‐fold higher secreted alkaline phosphatase (SEAP) activity than free cGAMP (*P* < 0.0001; Figure 2D). These data demonstrate that NFSA overcomes the two principal barriers to cGAMP translation: poor cell entry and inefficient ER delivery. Guanidinium‐rich MP2 peptides enhance membrane penetration and endosomal escape, thereby increasing cytosolic cGAMP availability and STING activation.

To ascertain whether the immunostimulatory effects of NFSA are specifically mediated through the STING pathway, we conducted a comparative analysis of the responses of wild‐type (WT) RAW 264.7 cells and STING1‐knockout (KO) RAW 264.7 cells following NFSA treatment. Initially, we quantified the secretion of downstream effector cytokines IP‐10 and IFN‐β ELISA kits (refer to Figure S10A). In WT cells, NFSA treatment elicited a significant and robust increase in the secretion of both IP‐10 and IFN‐β compared to free cGAMP. Conversely, in STING1‐KO cells, the NFSA‐induced cytokine release was nearly completely abolished, with secretion levels reverting to baseline.

To further elucidate the underlying molecular mechanism, we investigated the phosphorylation status of key proteins within the STING‐TBK1‐IRF3 signaling cascade through Western blot analysis (Figure S10B,C). In wild‐type (WT) cells, NFSA significantly enhanced the phosphorylation of STING, TBK1, and IRF3. Consistent with the ELISA findings, the phosphorylation of these critical signaling molecules induced by NFSA was completely abolished in STING1‐knockout (KO) cells (Figure S10C). The complete absence of both downstream kinase activation (p‐TBK1/p‐IRF3) and subsequent cytokine production in STING1‐deficient cells conclusively demonstrates that STING pathway functionality is essential for the immunomodulatory activity of NFSA.

In order to evaluate the conservation of the STING‐TBK1‐IRF3 signaling pathway activation and immunogenic cell death (ICD) across various neuroblastoma cell types, we employed human SH‐SY5Y and murine 9464D neuroblastoma cell lines. Additionally, the human THP‐1 monocyte/macrophage cell line was utilized as a control. Time‐course experiments were conducted to facilitate this assessment. Western blot analysis (Figure S11A–C) showed that NFSA treatment activated the STING pathway in all lines, though the activation was weaker in the neuroblastoma lines compared to THP‐1, as indicated by lower phosphorylation ratios of p‐TBK1/TBK1, p‐IRF3/IRF3, and p‐STING/STING. Despite this, phosphorylation kinetics were similar across all lines, with p‐STING, p‐TBK1, and p‐IRF3 levels peaking within 1 h and then declining synchronously. These findings suggest that the spatiotemporal pattern of “rapid activation and swift decay” in STING‐dependent downstream response triggered by NFSA is similarly observed in the neuroblastoma and immune cell models tested. To further confirm if activating the intracellular STING pathway leads to the release of extracellular immunogenic signals, we measured extracellular HMGB1 and intracellular ATP levels. Figure S11D showed that HMGB1 levels increased over time in all cell lines after NFSA treatment, while ATP levels dropped sharply between 0.5 and 1 h. This indicates effective DAMP release and early metabolic stress, signaling immunogenic death. The timing of peak STING phosphorylation and DAMP release highlights a closely linked signaling‐to‐death process, with quick activation and fast attenuation of the immune response aligning with the appearance of immunogenic markers in the examined cell lines.

### 3.3 Binding Affinity of NFSA to Human STING Protein

To quantitatively assess the direct molecular interaction between NFSA and human STING, surface plasmon resonance (SPR) technology was utilized. Serial dilutions of NFSA, ranging from 0.781 to 50 µm, were injected over immobilized, purified human STING protein, as characterized in Figure S13A. The sensorgrams, presented in Figure S13B, demonstrated a clear concentration‐dependent binding signal between NFSA and immobilized STING1. The binding profiles exhibited rapid association and relatively slow dissociation, indicative of the formation of a stable complex. Global fitting of the data was conducted using the Biacore Insight Evaluation Software with a 1:1 Langmuir binding model, resulting in ka of 5.01 × 10^2^ M^−^^1^s^−^^1^, kd of 1.99 × 10^−^^3^ s^−^^1^, and KD of 3.97 × 10^−^^6^ m (3.97 µm). The goodness of fit, as indicated by a Chi^2^ value of 7.31 × 10^−^^3^ RU^2^, confirmed the high quality of the fitting, as shown in Figure S13C.

Taken together, the SPR data collectively validate that the NFSA formulation, as an integrated supramolecular assembly, exhibits a concentration‐dependent binding interaction with immobilized human STING1 protein under the specified experimental conditions, demonstrating an apparent micromolar affinity. Considering that NFSA is composed of multiple elements—including the cGAMP cargo, the peptide backbone, and the resulting nanostructured topography—the observed binding reflects the comprehensive interaction profile of this multicomponent formulation, rather than being attributable to any single component. These quantitative SPR findings offer a biophysical characterization of the formulation–protein interaction, which may serve as a foundational reference for elucidating the downstream biological activities investigated in subsequent cellular and animal studies.

### 3.4 In Vitro Immunostimulatory Effects and TAM Repolarization of NFSA

To evaluate whether NFSA enhances dendritic cell antigen presentation, we analyzed the expression of maturation markers (CD80^+^ and CD86^+^) in BMDCs via flow cytometry. NFSA treatment significantly promoted BMDC maturation, with the proportion of CD80^+^CD86^+^ double‐positive cells increasing by 26.6% compared to the free cGAMP group (*p* = 0.0004, Figure 2E,F). ELISA revealed corresponding rises in IL‐6, IFN‐β and TNF‐α secretion to 767 ± 12, 364 ± 13 and 499 ± 82 pg mL^−^^1^, respectively (2.66‐, 1.42‐ and 11.1‐fold increases, all *p* < 0.0001, Figure 2H). These results indicate that NFSA stimulates BMDC maturation and pro‐inflammatory cytokine secretion via STING activation, which may reprogram the immunosuppressive tumor microenvironment.

To explore NFSA's ability to modulate immunosuppressive macrophages, we generated M0, M1, and M2‐like BMDM subsets using M‐CSF, LPS, IFN‐γ, and IL‐4. Compared to cGAMP alone, NFSA upregulated the M1 marker CD86 by 17.2% (*p* = 0.011) and downregulated the M2 marker CD206 by 10.5% (*P* < 0.0001, Figure 2G) in BMDMs, indicating reversal of immunosuppressive phenotypes. Concomitantly, IL‐6, IFN‐β and TNF‐α production rose to 1 266 ± 96, 30 ± 2 and 763 ± 38 Pg mL^−^^1^, respectively (1.9‐, 2.3‐ and 1.4‐fold Increases, all *p* < 0.0001, Figure 2I). We conducted an in‐depth analysis of the functional markers associated with tumor‐associated macrophage polarization, specifically focusing on nitric oxide (NO) and Arg‐1 (Figure S12). The findings indicated that M2‐Like BMDMs treated with PBS and PSNF exhibited a metabolic phenotype characteristic of the M2 type, marked by elevated Arg‐1 levels and reduced NO production, indicative of an immunosuppressive profile. In contrast, treatment with NFSA, as opposed to cGAMP, resulted in a marked decrease in Arg‐1 expression and secretion, alongside a significant increase in NO production. This shift promotes the transition of macrophages toward an M1‐type metabolic phenotype, which is associated with a pro‐inflammatory and anti‐tumor response. These findings demonstrate that NFSA promotes M1 polarization of BMDMs and enhances pro‐inflammatory factor expression, amplifying anti‐tumor immune responses.

To assess whether NFSA's regulates immunosuppressive macrophages in both murine BMDMs and human macrophage models, we differentiated THP‐1 cells into M0 macrophages and further polarized them into M1 and M2 phenotypes. Figure S14A showed THP‐1 cells initially in circular suspension. Upon differentiation into M0 macrophages using PMA, they become adherent with irregular shapes and larger volumes. M1 cells exhibit a polygonal, spread‐out morphology, while M2 cells have a slender, spindle‐like shape, confirming successful morphological polarization. Flow cytometry analysis provided a quantitative assessment of the impact of each treatment group on the polarization phenotype of THP‐1‐derived macrophages. As illustrated in Figure S14B–D, NFSA treatment, in comparison to free cGAMP, resulted in a significant increase of 11.37% in the proportion of M1‐type cells (CD68^+^CD86^+^) and a decrease of 17.9% in the proportion of M2‐type cells (CD68^+^CD206^+^). The degree of polarization averaged 1.30, indicating that the M1/M2 ratio was 2.7 times higher than that observed with cGAMP. Furthermore, the data presented in Figure S14E demonstrated that NFSA treatment led to a downregulation of IL‐4 and Arg‐1 expression in M2‐type macrophages, while significantly enhancing the secretion of pro‐inflammatory factors IL‐6 and NO in M1‐type macrophages. Notably, these trends were qualitatively consistent with the phenotypes observed in mouse BMDMs. Collectively, the findings suggest that comparable polarization‐modulating kinetics were observed in the human THP‐1 and murine BMDMs examined. This indicates that the regulatory influence of NFSA on the polarization of immunosuppressive macrophages is consistent across these cellular systems. However, further validation in primary cells or in vivo contexts is necessary.

### 3.5 In Vivo Therapeutic Efficacy and Immune Activation Mechanisms of Intratumoral NFSA in Murine Models

**FIGURE 3:**
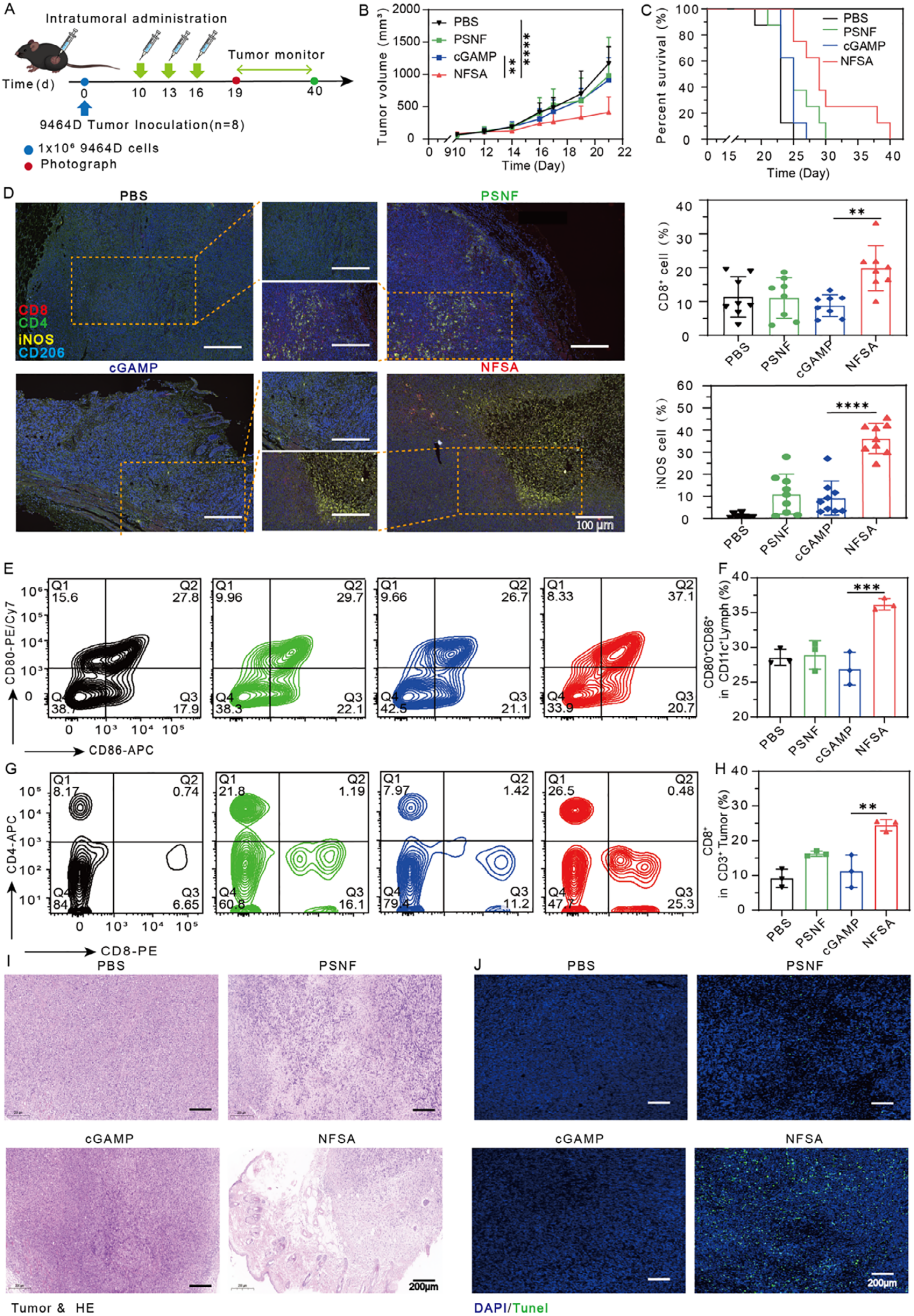
In vivo efficacy and immune activation effects of intratumoral NFSA in murine models. (A) Schematic of intratumoral administration. (B, C) Tumor growth and survival after intratumoral dosing (*n* = 8). (D) Representative IHC images of tumor tissues (scale bar = 100 µm) and quantification of intratumoral CD8^+^, CD4^+^, iNOS^+^ and CD206^+^ cells (*n* = 8). (E, F) Immune activation effects in lymph nodes of neuroblastoma‐bearing mice after intratumoral injection of different formulations. *n* = 3. (G, H) Immune activation effects in tumor tissues of neuroblastoma‐bearing mice after intratumoral injection of different formulations. *n* = 3. (I) Pathological changes in tumor sections of neuroblastoma‐bearing mice following intratumoral injection of different formulations.(Scale bar = 200 µm).(J) Apoptotic cell status in tumor sections of neuroblastoma‐bearing mice after intratumoral injection of different formulations.(Scale bar = 200 µm). The substantial variations in tumor growth among the groups were assessed utilizing ordinary one‐way analysis of variance. Data are shown as mean ± SD.*P < 0.05, **p < 0.01, ***p < 0.001, ****p < 0.0001.

Using a neuroblastoma (9464D) tumor‐bearing murine model, we systematically evaluated the in vivo antitumor efficacy of NFSA. Building on its validated in vitro immunostimulatory properties, we assessed its in vivo therapeutic potential to inform clinical translation. Following established intratumoral injection protocols for cGAMP (a widely used STING agonist), therapeutic efficacy was evaluated (Figure 3A). The PBS control group showed exponential tumor growth, while the PSNF and free cGAMP groups initially suppressed tumors, regrowth occurred over time, presumably due to rapid systemic drug clearance. Notably, NFSA group exhibited significantly superior tumor growth inhibition compared to the control groups (*p* = 0.0045, Figure 3B; Figure S16). Compared with the PBS, PSNF, and cGAMP groups, intratumoral injection of NFSA effectively prolonged the overall survival of tumor‐bearing mice in this group (Figure 3C).

Given the critical role of cytotoxic T lymphocyte (CTL) infiltration in immunotherapeutic responses, multiplex fluorescence immunohistochemistry (mIHC) and digital pathology were used to evaluate NFSA‐mediated modulation of key intratumoral immune subsets (CTLs, M1/M2‐TAMs; Figure S17; Figure 3D). Quantitative analysis across eight random fields demonstrated that NFSA significantly enriched tumor‐infiltrating CD8^+^ effector T cells (1.25‐fold vs cGAMP, *p* = 0.0014; Figure 3D) and CD4^+^ helper T cells (2.11‐fold, *p* = 0.0004, Figure S17). Immunofluorescence analysis of TAMs demonstrated that NFSA induces the polarization of macrophages towards the anti‐tumor M1 phenotype, as indicated by CD206^+^ and iNOS^+^ staining. NFSA enhanced the population of M1‐TAMs by a factor of 2.93 (*p* < 0.001; Figure 3D, Figure S15), aligning with the results observed in vitro during the polarization induction of BMDMs. This finding further substantiates NFSA's immunomodulatory activity in vivo.

Building on prior evidence of NFSA‐induced STING pathway activation, dendritic cell (DC) maturation, and antitumor activity (Figure 3A–D), we explored its systemic immunoregulatory mechanisms. Flow cytometric analysis of tumor‐draining lymph nodes showed significantly higher proportions of CD80^+^CD86^+^ double‐positive DCs in the NFSA group (36.2% ± 0.47%) versus the PBS (28.57% ± 0.66%), PSNF (28.93% ± 1.189%), and free cGAMP (26.93% ± 1.36%) groups (*p* = 0.004; Figure 3E,F; Figure S18). This confirms our in vitro findings, demonstrating that NFSA enhances DC maturation and antigen presentation to prime T cells for tumor targeting.

NFSA treatment increased tumor‐infiltrating CD8^+^ CTLs by 1.18‐fold (*p* = 0.0011) and CD4^+^ T cells by 3.18‐fold (*p* = 0.0004) compared to free cGAMP (Figure 3G,H; Figure S19A). Histopathological analysis revealed extensive necrosis and apoptotic bodies in NFSA‐treated tumors (Figure 3I), with TUNEL‐positive apoptotic rates 8.57‐fold higher than in the cGAMP group (*p* < 0.0001; Figure 3J), indicating STING‐driven immunogenic cell death synergizing with T cell cytotoxicity.

ELISA of serum cytokines showed that NFSA increased IFN‐β (3.29‐fold, *p* = 0.0238), IL‐6 (2.74‐fold, *p* = 0.0007), and TNF‐α (2.56‐fold, *p* = 0.0195) compared to free cGAMP (Figure S19B–D), highlighting STING‐mediated amplification of type I interferons and proinflammatory cytokine cascades.

Our findings elucidate NFSA's multifaceted mechanism of STING activation: enhancing DC‐mediated antigen presentation, inducing immunogenic tumor cell death, and triggering systemic release of inflammatory cytokines (IFN‐β, IL‐6, TNF‐α) to establish a positive immunoregulatory loop. These findings support the feasibility of peptide‐based STING agonist delivery and underscore localized intratumoral therapy as a strategy to orchestrate systemic antitumor immunity in pediatric solid tumors.

### 3.6 Antitumor Preventive/Therapeutic Effects, Tumor Targeting and Immune Activation of NFSA via Tail Vein Administration

Intratumoral (IT) injection has notable limitations: it is only applicable to solid tumors accessible for puncture, and long‐term, high‐frequency administration poses risks including chronic injection‐induced pain, disruption of the tumor microenvironment (TME) and vascular network, increased cancer cell extravasation and metastasis, and elevated incidence of infections and adverse reactions. These drawbacks have hindered the clinical translation of STING agonists. In contrast, intravenous (IV) administration offers advantages such as ease of operation and minimal tumor tissue damage, making it a widely used delivery method in clinical oncology. Thus, we investigated the antitumor preventive and therapeutic effects of NFSA via IV administration to evaluate its clinical translational potential.

**FIGURE 4:**
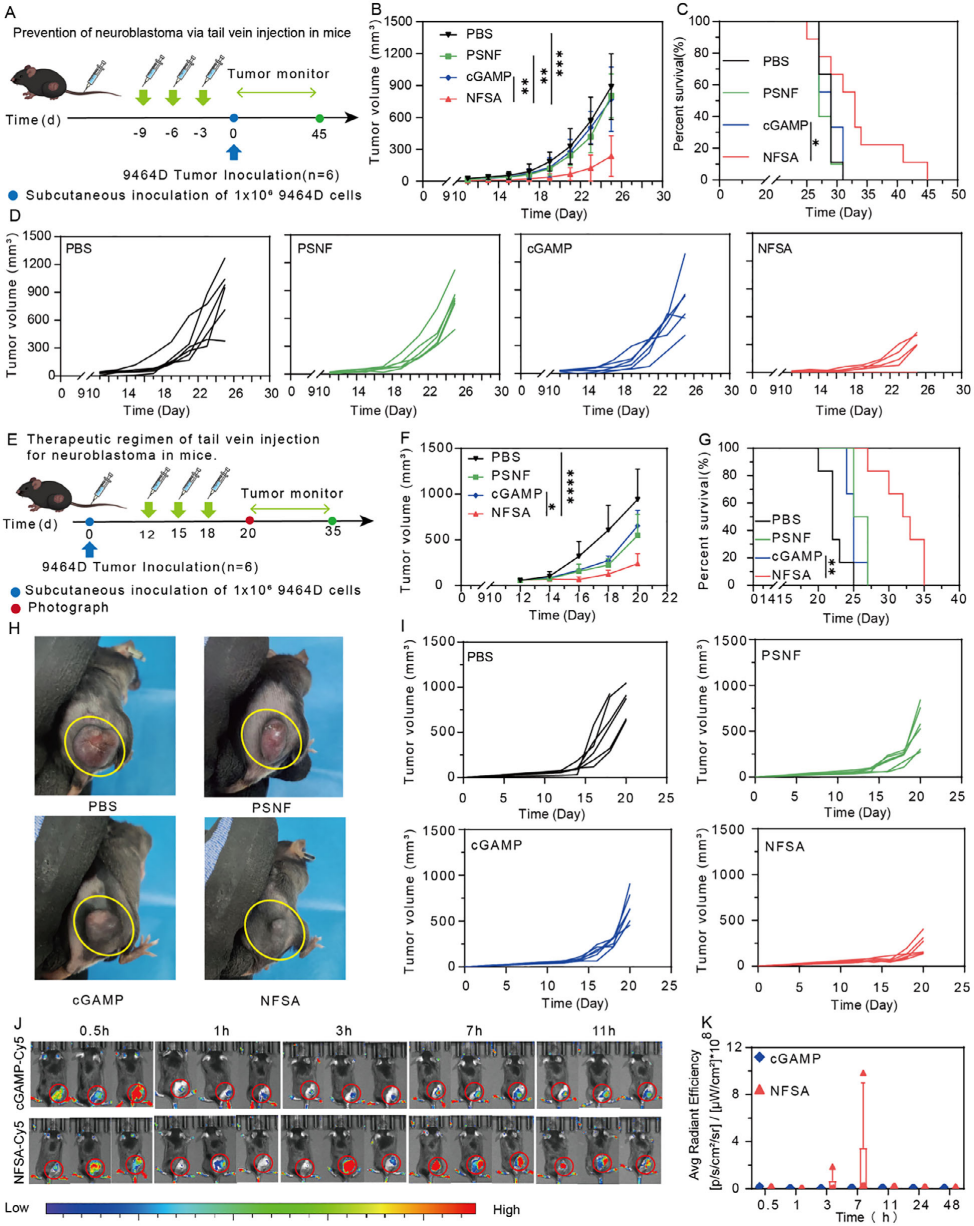
Study on the preventive efficacy, therapeutic efficacy in vivo and tumor targeting properties of NFSA administered via tail vein injection in neuroblastoma mice. (A) Schematic diagram of drug administration via tail vein injection in mice for the prevention of neuroblastoma. (B) Tumor volume changes in tumor‐bearing mice after injection with PBS, PSNF, cGAMP, and NFSA. (C) Survival curves of tumor‐bearing mice after injection with PBS, PSNF, cGAMP, and NFSA. (D) Individual tumor volume changes in each group after injection with PBS, PSNF, cGAMP, and NFSA. (E) Schematic diagram of a therapeutic administration regimen: treating neuroblastoma in mice via tail vein injection. (F, G) Tumor growth and survival after i.v. dosing (*n* = 6). (H) Changes in tumor volume in neuroblastoma mice following tail vein injection during the NFSA therapeutic efficacy assessment experiment. *n* = 6. (I) Changes in tumor volume in neuroblastoma mice following tail vein injection of PBS, PSNF, cGAMP, and NFSA during the NFSA therapeutic efficacy assessment experiment (*n* = 6). (J, K) In vivo biodistribution following i.v. injection (*n* = 3). The substantial variations in tumor growth among the groups were assessed utilizing ordinary one‐way analysis of variance. Data are mean ± SD; *p < 0.05, ****p < 0.0001.

In the antitumor prevention experiment, mice first received 3 doses of NFSA as prophylactic treatment, followed by inoculation with neuroblastoma cells. As shown in Figure 4A–D and Figure S20, tumor growth was significantly faster in the PBS, PSNF, and cGAMP groups, whereas it was markedly slower in the NFSA group. By approximately day 30, all mice in the PBS, PSNF, and cGAMP groups reached the ethical endpoint due to excessive tumor volume. In contrast, tumor progression in the NFSA group was retarded, with only two mice reaching the ethical endpoint by day 45 (Figure 4C). These results confirm NFSA's potential to inhibit tumor growth and prolong the survival of tumor‐bearing mice.

Subsequently, to evaluate therapeutic efficacy, 3 doses of NFSA were administered intravenously to tumor‐bearing mice pre‐inoculated with neuroblastoma cells (Figure 4E). Rapid tumor progression was observed in the PBS and free cGAMP groups—free cGAMP's low efficacy was presumably attributed to poor tumor targeting and limited tissue permeability. Although the PSNF group initially suppressed tumors, growth resumed uncontrollably in later stages. Notably, the survival period of the PBS group is the shortest, while the PSNF and cGAMP groups show a slightly longer survival period of the same length. The NFSA group exhibits the longest survival period among all groups. Specifically, compared with the cGAMP group, tail vein injection of NFSA results in a significant extension of the mice's survival period (Figure 4F–I), validating the therapeutic efficacy of IV‐administered NFSA.

To elucidate the mechanism underlying this efficacy, we assessed NFSA's in vivo biodistribution using an in vivo imaging system (IVIS). Analysis of tumor fluorescence intensity of cGAMP‐Cy5 showed that both free cGAMP‐Cy5 and NFSA‐Cy5 accumulated in tumors at 0.5 h post‐administration. However, free cGAMP‐Cy5 fluorescence decreased over time, whereas NFSA‐Cy5 intensity increased by 7 h and remained at approximately 40% of the initial level at 24 h (Figure 4J,K; Figure S21). This demonstrates that the peptide carrier enables NFSA to evade clearance, enhance tumor targeting and permeability, and prolong drug retention—key properties for precision cancer therapy.

To examine the interaction between NFSA nanofibers and the biological environment during systemic circulation, we performed a comprehensive proteomic analysis of the protein corona formed on the fiber surfaces. As illustrated in Figure S22A,B, the SDS‐PAGE bands and molecular weight distribution data reveal that the NFSA surface effectively adsorbed a substantial quantity of proteins. The molecular weights of these proteins predominantly ranged from 10 to 100 kDa, with notable distribution peaks observed at 30–50 kDa and 60–70 kDa.

Subsequent biological function annotation (refer to Figure S22C) indicated that while intracellular metabolic and structural proteins constituted 45.62% of the total, a significant enrichment of functional proteins was observed in the plasma. These included immune proteins (19.32%), complement proteins (9.23%), coagulation proteins (7.31%), apolipoproteins (5.8%), and acute phase proteins (11.27%) within the corona layer. As illustrated in Figure S22D, complement proteins emerged as the most prevalent components within the protein corona, with C3 being the most abundant at 8.61%, followed by immunoglobulins, where albumin was the most abundant at 7.19%. Regulatory molecules adsorbed onto the fiber surface are identifiable by complement receptors (CR3/CR4) located on TAMs and vascular endothelial cells, as well as Fcγ receptors. This interaction facilitates receptor‐mediated endocytosis, aiding the fibers in traversing the vascular barrier and enhancing the capture of immune cells within the tumor stroma, thereby extending their local retention time.

The protein corona was found to contain fibronectin (0.085%), vitronectin (0.16%), and transferrin (0.46%), which are integral components of the extracellular matrix (ECM). Upon adsorption onto the fiber surface, these proteins enable the nanofibers to “camouflage” themselves and establish high‐affinity interactions with integrin receptors on tumor and stromal cell surfaces, such as αvβ3 and α5β1. This binding not only provides robust physical anchoring, preventing the fibers from being displaced by blood flow or removed via lymphatic drainage, but also activates intracellular signaling pathways that promote the active retention of the fibers within the tumor stroma. The analysis of the aforementioned protein corona components indicates that the tumor accumulation mechanism of NFSA nanofibers is influenced not only by the passive EPR effect, but also by specific active targeting mechanisms.

### 3.7 Immune Activation Mechanisms and Clinical Translational Value of IV‐Administered NFSA

Given NFSA's antitumor efficacy, we further assessed its IV‐driven immune activation and clinical potential by systematically analyzing the remodeling of the tumor immune microenvironment (TIME), in comparisons to IT injection.

Building upon the evaluation of the therapeutic efficacy of intravenously administered NFSA as previously described, we conducted an immunohistochemical (IHC) analysis on ex vivo lymphoid tissues to characterize the immune phenotypes present within these tissues (Figure 4H). Quantitative analysis of eight randomly selected high‐power fields (HPFs) revealed that, in comparison to the cGAMP group, NFSA treatment resulted in a twofold increase in the proportion of CD8^+^ cytotoxic T cells (*p* = 0.0053), while slightly reducing the proportion of CD4^+^ T cells (Figure S23). This administration of NFSA led to a significant elevation in the CD8^+^/CD4^+^ ratio within the lymphoid tissues, suggesting an increased relative predominance of cytotoxic T lymphocytes among the T cell subsets at the phenotypic level.

**FIGURE 5:**
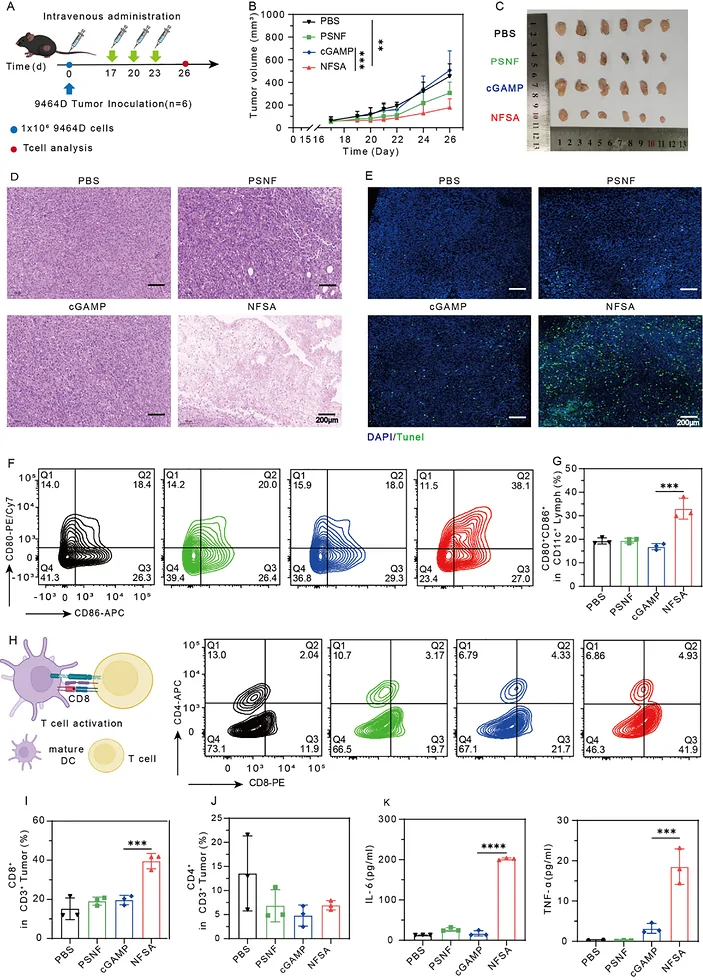
Study on immune activation effects in mice via tail vein administration. (A) Schematic diagram of tail vein injection for neuroblastoma‐bearing mice. (B, C) Tumor volume changes in neuroblastoma‐bearing mice after tail vein injection of different formulations. *n* = 6. (D) Pathological changes in tumor sections of neuroblastoma‐bearing mice after tail vein injection of different formulations. (Scale bar = 200 µm). (E) Cell apoptosis in tumor sections of neuroblastoma‐bearing mice after tail vein injection of different formulations. (Scale bar = 200 µm). (F, G) Lymph node immune activation effects in neuroblastoma‐bearing mice after tail vein injection of different formulations. (H–J) Schematic diagram of mature dendritic cells (mature DC) activating T cells into cytotoxic T lymphocytes (CD8^+^T cell) via antigen presentation and tumor tissue immune activation effects in neuroblastoma‐bearing mice after tail vein injection of different formulations. *n* = 3. (K) Secretion levels of cytokines IL‐6 and TNF‐α in neuroblastoma‐bearing mice following tail vein injection of different formulations. *n* = 3. The substantial variations in tumor growth among the groups were assessed utilizing ordinary one‐way analysis of variance Data are shown as mean ± SD. *P < 0.05, **p < 0.01, ***p < 0.001, ****p < 0.0001.

Subsequently, to further dissect NFSA's immune‐regulatory effects at the tissue and systemic levels, we administered three doses of NFSA via IV to tumor‐bearing mice. After treatment, we harvested lymph nodes and tumor tissues for immune cell profiling and collected serum samples for cytokine analysis. Experimental results evaluating the in vivo immune‐activating effects of NFSA showed rapid tumor growth in the PBS and free cGAMP groups. Although the PSNF group initially exhibited tumor inhibitory effects post‐administration, tumor growth became uncontrollable in the later stages. Notably, the IV NFSA group showed significant suppression of tumor growth (Figure 5A–C, Figure S24). Hematoxylin and eosin (H&E) staining revealed larger necrotic areas in NFSA‐treated tumors (Figure 5D), and TUNEL apoptosis assays confirmed that the IV NFSA group had significantly higher levels of tumor cell apoptosis compared to other groups (*p* < 0.0001, Figure 5E). These results further confirm that IV‐administered NFSA exhibits favorable therapeutic efficacy.

Given that dendritic cell (DC) maturation and antigen presentation are pivotal for initiating adaptive antitumor immunity, we used flow cytometry to quantify CD80^+^CD86^+^ double‐positive DCs (a well‐recognized marker of mature DCs) in lymph nodes (Figure 5E,F). Flow cytometry analysis (Figure 5F,G) showed that IV‐administered NFSA increased the proportion of CD80^+^CD86^+^ DCs in lymph nodes to 33.09 ± 2.557%, significantly higher than that in the PBS (19.3 ± 0.755%, *p* = 0.0004), PSNF (19.47 ± 0.584%, *p* = 0.0004), and free cGAMP groups (16.9 ± 0.737%, *p* = 0.0001). Remarkably, this proportion was comparable to that in the IT group (36.2 ± 0.47%, p = 0.292), confirming that IV administration effectively promotes DC maturation and overcomes the targeting limitations of traditional STING agonist delivery.

To characterize T‐cell subsets (CD8^+^ and CD4^+^) in tumor tissues, we performed flow cytometry (Figure 5H–J). Results demonstrated that CD8^+^ cytotoxic T‐cell infiltration in the IV NFSA group reached 39.53 ± 2.267%, twice that of the cGAMP group (19.67 ± 1.393%, *p* = 0.0005), while the proportion of CD4^+^ helper T cells was 6.93 ± 0.558%, slightly lower than that in the other three groups. This suggests that IV administration of the NFSA peptide formulation may primarily exert its antitumor immunotherapeutic effects by promoting increased CD8^+^ T‐cell infiltration in mice.

ELISA analysis (Figure 5K) showed elevated serum cytokine levels in the IV NFSA group: interleukin‐6 (IL‐6) reached 201.8 ± 1.351 pg mL^−1^ (10.7‐fold higher than the cGAMP group, *p* < 0.0001), and tumor necrosis factor‐α (TNF‐α) reached 18.55 ± 2.542 pg mL^−1^ (5.7‐fold higher than the cGAMP group, *p* = 0.0001). These results confirm that NFSA triggers cascade activation of antitumor signaling pathways, where synergistic cytokine amplification enhances systemic immunotherapeutic efficacy.

Collectively, this study demonstrates that IV‐administered NFSA achieves antitumor efficacy comparable to IT injection by integrating systemic and local effects: elevated peripheral pro‐inflammatory cytokine responses and localized TIME remodeling form a positive feedback loop that amplifies antitumor immunity. This finding overcomes a key limitation of current STING agonist‐based therapies, which traditionally rely on localized delivery, thus providing critical experimental evidence for the development of IV‐based tumor immunotherapies. Notably, by addressing clinical scenarios where IT injection is impractical (e.g., for deep‐seated or metastatic tumors), the NFSA formulation markedly improves translational potential and supports its utility in broader oncology applications.

### 3.8 Immune Serological Verification and Its Transformation Safety

To assess the humoral immunogenicity of NFSA and its transformation safety, we quantitatively measured the specific antibody responses in the serum of mice against MP1 and MP2. MP1 and MP2 were conjugated to the carrier protein BSA, as illustrated in Figure S25A,B, which also includes their molecular weights. Multiple booster immunizations were administered to C57BL/6 mice to generate positive sera. The specific antibody levels in the mouse sera were determined using ELISA, with results depicted in Figure S25C. The antibody titer against MP1 reached 1:2133.333333, while the titer against MP2 reached 1:600. Figure S25D presents the OD values of anti‐MP1/MP2 antibodies across four groups (PBS control, PSNF, cGAMP, NFSA) at intervals of 3, 7, 14, 30, and 60 days post‐administration. The antibody levels in the PSNF and NFSA groups peaked rapidly on day 7; however, although their OD values were similar, they consistently remained below the predetermined critical threshold (OD = 0.263). Subsequently, the antibody levels declined rapidly. By the 14th day, a significant reduction in antibody levels was observed. Furthermore, between the 30th and 60th days, these levels reverted to baseline, demonstrating no statistically significant difference from the negative control group. The findings indicate that NFSA demonstrates robust dynamic safety and offers compelling evidence supporting the biological safety of the platform. This is particularly noteworthy as it effectively mitigates the significant risks associated with long‐term immune complex deposition or chronic hypersensitivity reactions in pediatric pharmacotherapy.

### 3.9 Biosafety Evaluation of NFSA

Finally, we systematically validated its biosafety across in vitro and in vivo models, including biocompatibility, acute toxicity, and organ‐specific toxicity.

**FIGURE 6:**
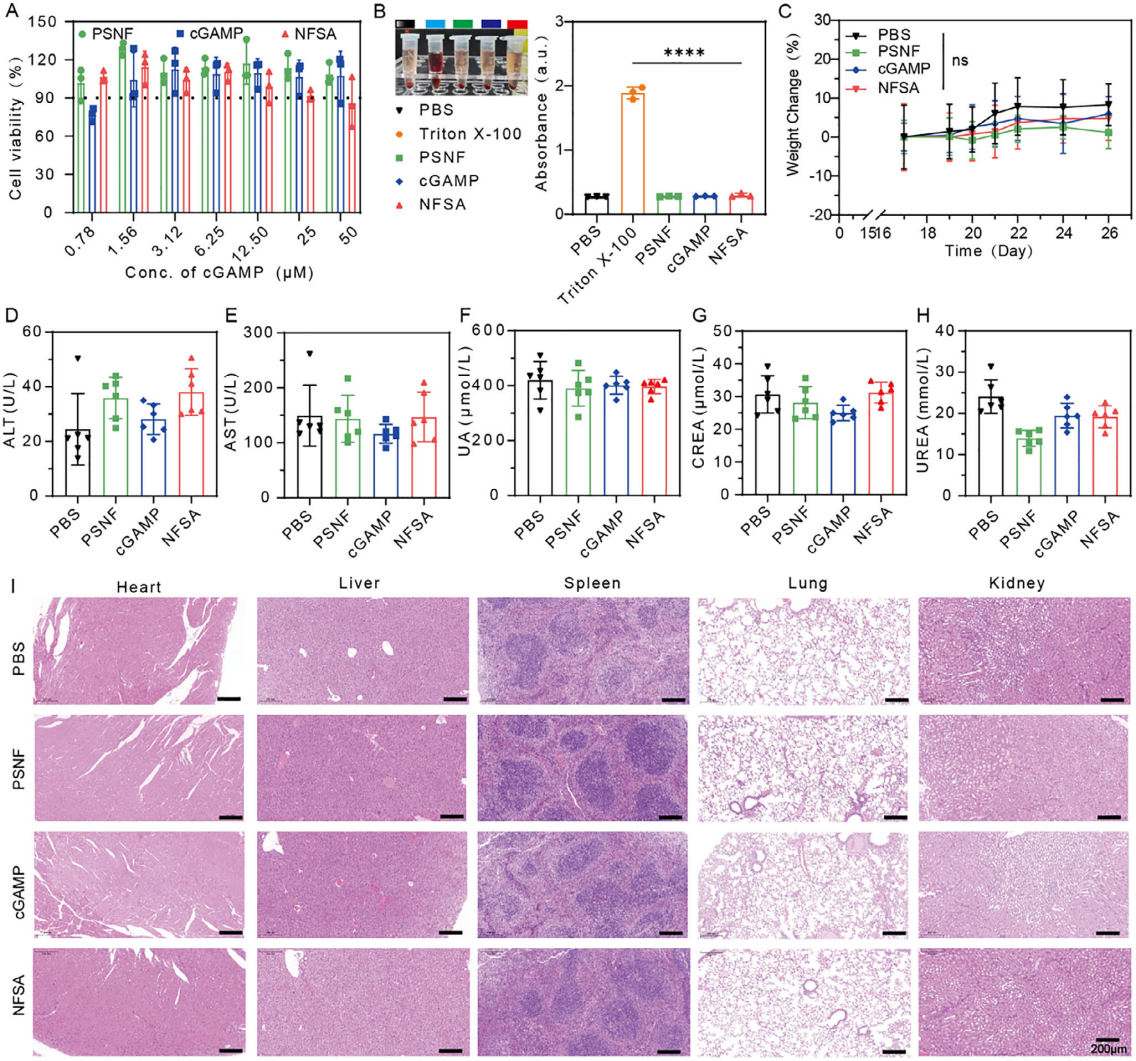
Biosafety evaluation of NSFA. (A) In vitro cellular toxicity of polypeptide formulations (*n* = 3). (B) In vitro hemocompatibility of polypeptide formulations (*n* = 3). (C) Effect of tail vein‐administered polypeptide formulations on mouse body weight changes. (D–H) Hepatic and renal function after tail vein administration (*n* = 6). (I) H&E staining images of major tissue sections post‐intravenous delivery (*n* = 3; scale bar = 200 µm). Data are presented as mean ± SD. *p < 0.05, **p < 0.01, ***p < 0.001, ****p < 0.0001.

Cellular toxicity was evaluated using RAW264.7 macrophages (Figure 6A). After 24‐hour treatment with 50 µM NFSA, cell viability remained at 86.03%, with no significant difference from the blank control (*p* = 0.1057). For blood compatibility, the hemolysis rate in the NFSA group was 1.769 ± 1.812%, significantly lower than the Triton X‐100 positive control (100 ± 3.293%, *p* < 0.0001) (Figure 6B). This meets the ISO 10993‐5 safety threshold (<5%), indicating stability in blood and minimal risk of coagulation or thrombosis.

In neuroblastoma‐bearing mice, body weight fluctuations after intravenous (IV) or intratumoral (IT) NFSA administration showed no significant difference from the PBS control over 24 d (*p* = 0.45), with no acute toxicity symptoms (e.g., reduced food intake or activity) observed (Figure 6C; Figure S26).

Blood biochemical analysis post‐treatment revealed normal levels of alanine aminotransferase (ALT), aspartate aminotransferase (AST), creatinine (CREA), blood urea nitrogen (UREA), and uric acid (UA) compared to controls (Figure 6D–H; Figure S26), confirming no systemic toxicity.

H&E staining of major organs (heart, liver, spleen, lungs, kidneys) showed intact structures without abnormalities: hepatic lobules without inflammation/steatosis, normal glomeruli and renal tubules, aligned myocardial fibers, clear alveoli, and distinct splenic red/white pulp boundaries. Organ architectures in all treatment groups were indistinguishable from controls (Figure 6I; Figure S26).

NFSA exhibits excellent biosafety with low cell toxicity, favorable blood compatibility, and no acute/chronic organ damage. These attributes support its potential as a safe nanodelivery platform for IV/IT administration, facilitating future clinical translation.

## 4 Discussion

The rational design of NFSA is based on the structural mimicry of cGAMP–STING interactions. By incorporating essential STING‐binding residues into amphiphilic peptides and further functionalizing them with a hydrophobic Benz‐FFF‐G motif, we developed a supramolecular system capable of multivalent, non‐covalent interactions with cGAMP. This biomimetic approach facilitates specific cargo recognition and stabilizes the nanofiber architecture, thereby distinguishing NFSA from traditional CDN carriers that depend on nonspecific electrostatic encapsulation.

A significant advancement of NFSA is its dual role as both a delivery vehicle and a bioactive modulator. The guanidinium‐rich MP2 sequence imparts intrinsic cell‐penetrating and endosomal‐escape capabilities, effectively overcoming the primary pharmacological challenges associated with STING agonists: limited membrane permeability and inefficient cytosolic delivery. This results in a 25.8‐fold increase in cellular uptake and enhanced localization to the ER, while the caveolae‐mediated entry pathway facilitates lysosomal avoidance. Additionally, the pH‐responsive conformational transition of the nanofibers—from α‐helix to β‐sheet/random coil under acidic tumor conditions—initiates localized drug release, thereby reducing systemic exposure and enhancing tumor‐specific bioavailability. Notably, the murine and human cell‐line models showed similar phosphorylation kinetics in the STING‐TBK1‐IRF3 pathway and comparable levels of immunogenic cell death markers (HMGB1, ATP), indicating a consistent response to cGAMP‐mediated signaling across these systems.

In vivo studies have demonstrated that NFSA exhibits significant antitumor efficacy through both intratumoral and intravenous administration, findings that hold substantial translational importance. The intravenous delivery, which presents a significant clinical challenge for STING agonists, was facilitated by a beneficial protein corona composition. This composition supports receptor‐mediated transcytosis and integrin‐mediated stromal retention, thereby transforming passive enhanced permeability and retention effects into active tumor targeting. NFSA treatment markedly promoted dendritic cell maturation, M1 macrophage polarization, and CD8^+^ T‐cell infiltration, while also inducing immunogenic cell death and systemic cytokine amplification. This establishes a positive immunoregulatory feedback loop that reconfigures the immunosuppressive tumor microenvironment. Comprehensive biosafety assessments—including cell viability (86.03% at 50 µm), hemocompatibility (hemolysis rate of 1.77%, below the ISO 10993–5 threshold), normal serum biochemical parameters, unremarkable histopathological findings in major organs, and a rapid decline of anti‐peptide antibody titers to baseline within 30–60 d—demonstrate that NFSA possesses a favorable safety profile, making it suitable for translational development.

Despite these advancements, several limitations necessitate acknowledgment. Firstly, the in vivo efficacy studies were conducted using immunocompetent murine neuroblastoma models; therefore, extrapolation to human tumors, which typically display greater genetic heterogeneity and more complex immunosuppressive microenvironments, has yet to be established. In this context, although THP‐1‐derived macrophages offer preliminary insights into human cellular responses, they do not fully replicate the characteristics of primary human monocyte‐derived macrophages. Therefore, our findings, especially those pertaining to human translational potential, should be approached with caution. It is imperative that these results be validated in primary human immune cells as a crucial subsequent step. Secondly, although the protein corona analysis offers mechanistic insights into tumor accumulation, further elucidation is required regarding the dynamic evolution of corona composition over time and its impact on pharmacokinetics. Thirdly, the optimal dosing schedule and the potential for combination with immune checkpoint inhibitors—a strategy that could further enhance the observed CD8^+^ T‐cell responses—warrant systematic investigation. Lastly, the long‐term biodistribution and clearance pathways of the peptide nanofibers, particularly at higher doses or with repeated administration schedules, require comprehensive evaluation.

## 5 Conclusion

In this study, we have engineered a biomimetic peptide‐based supramolecular nanocarrier (NFSA) designed for the efficient cytosolic delivery of the STING agonist cGAMP. This design was informed by the structural characteristics of the STING–cGAMP binding interface. The self‐assembled nanofibers demonstrate high drug encapsulation efficiency, pH‐responsive release, and enhanced membrane penetration through guanidinium‐rich motifs, effectively addressing the challenges of limited cellular uptake and inefficient endosomal escape. Mechanistically, the NFSA‐mediated delivery of cGAMP activates the STING–TBK1–IRF3 signaling pathway, facilitates dendritic cell maturation, and reprograms immunosuppressive tumor‐associated macrophages to an M1‐like phenotype, thereby inducing robust innate and adaptive antitumor immune responses in both murine and human cell models. The employment of STING1‐knockout cells corroborates that these immunostimulatory effects are contingent upon STING, consistent with the established role of the administered cargo, cGAMP. Notably, the intravenous administration of NFSA demonstrates antitumor efficacy comparable to that of intratumoral injection, characterized by extended tumor retention, advantageous protein corona‐mediated active targeting, and excellent biosafety profiles as evidenced by cellular, hematological, and histopathological evaluations. The low immunogenicity of the peptide components further underscores its appropriateness for repeated administration. Collectively, this study establishes NFSA as a promising nanocarrier platform for pathway modulation, particularly in cases where localized delivery is impractical. It also proposes a generalizable strategy for the design of self‐assembled peptide nanomedicines that integrate innate immune activation with precision oncology. Nonetheless, we recognize the need for further validation in primary human immune cells and a more comprehensive mechanistic analysis of the nanocarrier's interaction with STING to fully ascertain its translational potential.

## Author Contributions

Dong Mei, Yubin He, Xiao Lu, Ran Zhang, and Bo Wan designed the experiments. Yubin He and Xiao Lu performed the experiments, analyzed the data, and drafted the manuscript. Dong Mei and Xin Ni edited the manuscript. All authors reviewed and approved the final version.

## Conflicts of Interest

The authors declare no conflicts of interest.

## Supporting information




**Supporting File**: advs77967‐sup‐0001‐SuppMat.docx.

## Data Availability

The data that support the findings of this study are available from the corresponding author upon reasonable request.
