# Supplementary material for: Engineered Peptide Self‐Assembled Nanofibers for Enhanced STING Activation: A Promising Nanodelivery Platform for Immunotherapy of Pediatric Solid Tumors
Source: Adv Sci (Weinh). 2026 Sep 27:e77967. Online ahead of print. doi: 10.1002/advs.77967 (PMC13616383; doi:10.1002/advs.77967)
Supplement: Supplementary file 1 [file ADVS-9999-e77967-s001.docx]

**Supporting Information for ORIGINAL ARTICLE**

**Engineered Peptide Self-Assembled Nanofibers for Enhanced STING Activation: A Promising Nanodelivery Platform for Immunotherapy of Pediatric Solid Tumors**

Yubin He^a,^^d†^, Xiao Lu^a^^†^，Chang qing Yang^d^, Ran Zhang^a,d^, Bo Wan^a,d^, Xin Ni^c^*, and Dong Mei^a, b^*

Affiliation

a Department of Pharmacy, Beijing Children’s Hospital, Capital Medical University, National Center for Children’s Health, Beijing 100045, China

b Clinical Research Ward, Capital Center for Children's Health, Capital Medical University, Capital Institute of Pediatrics, Beijing 100020, China

c National Center for Pediatric Cancer Surveillance, Beijing Children’s Hospital, Capital Medical University, National Center for Children’s Health, Beijing 100045, China

d School of Basic Medicine and Clinical Pharmacy, China Pharmaceutical University, Nanjing 211198, China.

^†^These authors contributed equally to this work.

*Corresponding authors.

These authors made equal contributions to this work.

Figure S1 The molecular structures of 2',3'-cGAMP,modified peptides MP1 and MP2.

Figure S2 The structural basis of cGAMP binding to STING protein and the selection principle of two specific peptide chains (P1 and P2). (A-B) Schematic diagram and sequence alignment of cGAMP binding to STING protein, the structure is from the PDB protein database (https://www.rcsb.org/), PDB ID: 4KSY; red and purple circles represent the two specific binding peptide chains P1 (SYYIGY) and P2 (RAGIKDRVYS), respectively; yellow and blue marked amino acids indicate the specific binding sites of STING protein to cGAMP, the GI of Homo sapiens and Mus musculus are 119582502 and 254692993, respectively. (C) Molecular docking diagrams of MP1 and MP2 binding to cGAMP, respectively. (D) Mass spectrum of MP1 : Benz-FFF-G-SYYIGY. (E) Mass spectrum of MP2 : Benz-FFF-G-RAGIKDRVYS.

| Formula-tion ID | cGAMP（µM） | Proportion | Diameter（nm） | PDI | EE(%) |
| --- | --- | --- | --- | --- | --- |
| MP1SA | 7.4 | cGAMP:MP1 = 4:25 | 6.91 ± 0.58 | 0.19 ± 0.003 | 38.99 ± 8.17 |
| MP2SA | 7.4 | cGAMP:MP2 = 4:25 | 7.78 ± 1.62 | 0.095 ± 0.059 | 45.78 ± 13.4 |
| NFSA-1 | 7.4 | cGAMP:MP1:MP2 = 4:21:7 | 19.24 ± 0.34 | 2.01 ± 0.018 | 28.36 ± 5.22 |
| NFSA-2 | 7.4 | cGAMP:MP1:MP2 = 4:20:4 | 15.40 ± 0.88 | 0.12 ± 0.034 | 50.12 ± 2.47 |
| NFSA-3 | 7.4 | cGAMP:MP1:MP2 = 4:20:5 | 11.72 ± 2.85 | 0.661 ± 0.35 | 74.33 ± 1.52 |
| NFSA-4 | 7.4 | cGAMP:MP1:MP2 = 4:20:5（UF） | 8.99 ± 0.23 | 0.05 ± 0.008 | 81.09 ± 0.22 |

Table S1 The ratio relationships of MP1, MP2, and cGAMP in different formulations, along with their particle size, PDI (polydispersity index) dispersion coefficient,

and encapsulation efficiency. Data are shown as mean ± SD.

Figure S3 (A) Appearance of solution preparations from different prescriptions. (B) The TEM morphology of self-assembled polypeptide nanostructures at pH 5.2. (Scale bar = 100 nm).

Figure S4 (A) Fluorescence intensity and (B) cumulative release rate of cGAMP-Cy5 from NFSA at different times at pH 6.5. (C) The particle size of NFSA was measured at different times at pH 6.5. (D) The particle size and zeta potential of NFSA were measured in PBS over a period of 72 h. Data are presented as mean ± SD ( n = 3).

Figure S5 (A) The particle size and (B) zeta potential of NFSA were analyzed in a 10% serum environment over a period of 72 h. (C) Qualitative analysis of the protein corona components adsorbed by NFSA. The protein bands were visualized by Coomassie staining. Data are presented as mean ± SD ( n = 3).

Figure S6 Confocal microscopy was utilized to observe the cellular uptake efficiency of cGAMP-Cy5 and NFSA-Cy5 in RAW264.7, 9464D, and DC 2.4 cells.(Scale bar = 50 µm).

Figure S7 (A) Mass spectrum and HPLC of MP1’: Benz-FFF-G-YGYISY. (B) Mass spectrum and HPLC of MP2’: Benz-FFF-G-RAGIKDRVYS. (C) Representative particle sizes, zeta potentials, and TEM images of Ctrl-NFSA. Scale bar = 100 nm. Data are presented as mean ± SD ( n = 3).

Figure S8 Intracellular localization of free cGAMP-Cy5 (A), NFSA-Cy5 (B), and Ctrl-NFSA-Cy5 (C) in DC 2.4 cells after 0.5, 2, and 4 h of incubation. Purple and yellow signals indicate cGAMP-Cy5 colocalization with lysosomes and the endoplasmic reticulum, respectively. Scale bar = 1 nm.

Figure S9 (A) The uptake and (B) inhibition rates of NFSA by RAW264.7 cells treated with CPZ, AMI, and GEN were assessed using flow cytometry analysis. Data are presented as mean ± SD ( n = 3).

Figure S10 (A) The secretion of IP-10 and IFN-β were further measured by ELISA assays 8 h after receiving different treatments in both wild-type (WT) RAW 264.7 cells and STING1-Knockout (KO) RAW 264.7 cells (n = 3). (B) Western blot analysis of STING pathway-associated proteins in both wild-type (WT) RAW 264.7 cells and STING1 Knockout (KO) RAW 264.7 cells. (C) Quantitative results of (B) (n = 2). Data are shown as mean ± SD, **P* < 0.05, ***P* < 0.01, ****P* < 0.001, *****P* < 0.0001.

Figure S11 (A) Western blot analysis of STING pathway-associated proteins in SH-SY5Y cells, 9464D cells, and THP-1 cells at different time points. (B) Quantitative results of STING pathway-associated proteins in SH-SY5Y cells and 9464D cells (C) Quantitative results of STING pathway-associated proteins in THP-1 cells. (D) Extracellular HMGB1 levels and intracellular ATP levels in SH-SY5Y cells, 9464D cells, and THP-1 cells. HMGB1 in the supernatants was measured by ELISA, and intracellular ATP was detected using a bioluminescence assay. n = 2, Data are shown as mean ± SD.

Figure S12 (A) The secretion levels of NO and Arg-1 were measured by ELISA after treatment of M2-like BMDMs with PBS, PSNF, cGAMP, and NFSA for 24 h. n = 6 (B) Representative Western blot images of BMDMs treated with PBS, PSNF, cGAMP, and NFSA for 24 h. The expression level of Arg-1 protein expression analyzed by Western blotting. n = 2. Data are shown as mean ± SD, **P* < 0.05, ***P* < 0.01, ****P* < 0.001, *****P* < 0.0001.

Figure S13 (A) The SDS-PAGE profile of the full-length human STING1 protein. (B) SPR kinetics of the interaction between NFSA (concentrations ranging from 0.781 to 50 μM) and immobilized STING1. The red lines represent the global fitting curves using a 1:1 Langmuir binding model. (C) Steady-state binding affinity plot between NFSA and immobilized STING1.

Figure S14 (A) Representative bright-field images showing the morphological features of undifferentiated THP-1 cells and THP-1-derived macrophages polarized into M0, M1, and M2 phenotypes. Scale bars, 200 µm. Flow cytometry was used to analyze and identify the expression of M1 and M2 macrophages in each group. Representative flow cytometry results (B), quantitative statistics (C), and the M1/M2 macrophage ratio (D) were presented. (E) ELISA was employed to measure cytokine expression in M1 and M2 macrophages. n = 3. Data are shown as mean ± SD, **P* < 0.05, ***P* < 0.01, ****P* < 0.001, *****P* < 0.0001.

Figure S15 Immunofluorescence staining of the tumor slices after different treatments (Nucleus, blue fluorescence; M1 (iNOS), red fluorescence; M2 (CD206), green fluorescence). Scale bar = 50 µm.

Figure S16 (A-B) Changes in tumor volume in tumor-bearing mice following intratumoral injection in the NFSA tumor treatment efficacy study. n=8.

Figure S17（A-B）Quantitative results of immunohistochemical staining for CD4^+^ T cells and CD206^+^ cells in tumor tissue from mouse models of neuroblastoma treated with intratumoral NFSA injection.

Figure S18 (A) Schematic diagram of intratumoral injection administration protocol in vivo immunostimulation effect experiment using neuroblastoma-bearing mice. (B-D)Changes in tumor volume in tumor-bearing mice following intratumoral injection in vivo immune activation efficacy study. n=6.Data are shown as mean ± SD. **P* < 0.05, ***P* < 0.01, ****P* < 0.001, *****P* < 0.0001.

Figure S19 (A) In vivo immune activation study of neuroblastoma-bearing mice treated with intratumoral injections of different therapeutic agents, flow cytometry was performed to analyze the immunostimulatory effects of CD4⁺ T cells within tumor tissues.n = 3. Data are shown as mean ± SD.**P* < 0.05, ***P* < 0.01, ****P* < 0.001, *****P* < 0.0001.（B-D）In the experiment investigating the in vivo immune activation effect of intratumoral injection in neuroblastoma-bearing mice, the secretion levels of cytokines IFN-β (B), IL-6 (C), and TNF-α (D) were measured after different drug administration treatments.n = 3. Data are shown as mean ± SD.**P* < 0.05, ***P* < 0.01, ****P* < 0.001, *****P* < 0.0001.

Figure S20 In the experiment of preventing tumor occurrence and development via tail vein drug injection, the changes in tumor volume of tumor-bearing mice were observed after different drug administration treatments. n=6.

Figure S21 In vivo mouse imaging experiment, the distribution of cGAMP-Cy5 and NFSA-Cy5 in neuroblastoma-bearing mice at 24h and 48h after tail vein injection.

n = 3.

Figure S22 Compositional analysis of the protein corona adsorbed onto the NFSA surface. (A) Coomassie blue staining showing the molecular weight distribution of the adsorbed proteins. (B) Classification of the protein corona components based on calculated molecular weight according to proteomic results. (C) Classification of the protein corona components based on biological functions according to proteomic results. (D) Identification and presentation of key proteins associated with the tumor accumulation mechanism. Data are presented as mean ± SD ( n = 3).

Figure S23 (A) In the experiment of treating neuroblastoma - bearing mice via tail - vein drug injection, the immunohistochemical staining images of the mice's lymph nodes. (B)Quantification of IHC of CD8⁺ and CD4⁺ T cells in lymph nodes (n = 8). Scale bar = 100 µm.

Figure S24 In the experiment evaluating the in vivo immune activation effects of NFSA, tumor volume changes were measured in each neuroblastoma mouse following tail vein injection. n=6.

Figure S25 (A) Mass spectrum of Cys-MP1; (B) Mass spectrum of Cys-MP2; (C) Levels of specific antibodies to MP1 and MP2 in mouse serum. (D) The absorbance values of anti-MP1/MP2 in mouse serum were measured at various time points following treatment. Data are presented as mean ± SD ( n = 6).

Figure S26 In the intratumoral injection immune activation experiment,biosafety evaluation of Intratumoral Injection of Polypeptide Nanomedicine. (A) Impact on body weight changes in mice after intratumoral injection. n = 6. (B-F) Hepatorenal safety assessment following intratumoral administration.n = 6. (I) Hematoxylin and eosin (HE) staining: Visceral organ safety evaluation post intratumoral injection. n = 3. Data are shown as mean ± SD.
